# TOGR3, a Proteasome β4 Subunit, Orchestrates Sugar Homeostasis to Trade Off Growth and Thermotolerance in Rice

**DOI:** 10.1002/advs.202516395

**Published:** 2026-01-21

**Authors:** Biyao Zhang, Xiaoyan Wu, Ting Xu, Feifei Guo, Xiaolu Shen, Cuiping Meng, Yanan Wang, Xue Han, Hong Zhao, Yongbiao Xue

**Affiliations:** ^1^ Laboratory of Advanced Breeding Technology Institute of Genetics and Developmental Biology Chinese Academy of Sciences Beijing China; ^2^ National Genomics Data Center China National Center for Bioinformation Beijing China; ^3^ Beijing Institute of Genomics Chinese Academy of Sciences Beijing China; ^4^ University of Chinese Academy of Sciences Beijing China

**Keywords:** 26S proteasome subunit, sugar homeostasis, stomata, thermoresponsive growth, thermotolerance, TOGR3, TT1

## Abstract

Balancing growth and stress resilience remains a central challenge in breeding stress‐resistant crops. Here, we identify *TOGR3* (*THERMOTOLERANT GROWTH REQUIRED 3*), which encodes the rice 26S proteasome β4 subunit, as a key regulator coordinating these two processes. Ubiquitylome profiling reveals that the TOGR3‐dependent ubiquitin‐proteasome system (UPS) maintains sugar homeostasis by selectively controlling the turnover of sugar‐metabolizing enzymes. This regulation promotes leaf sugar accumulation, which serves dual roles by supplying carbon to sustain growth and by optimizing stomatal development and aperture dynamics to enhance leaf cooling capacity. Together, these mechanisms synergistically promote thermal‐adaptive growth and thermotolerance in rice. Notably, combined overexpression of *TOGR3* with the α2 subunit gene *TT1* further enhances heat resistance, underscoring the potential of coordinated proteasome subunit engineering for crop improvement. Our findings uncover a regulatory module that fine‐tunes the trade‐off between plant growth and abiotic stress resilience, providing promising molecular targets for breeding climate‐resilient crops.

## Graphical abstract

This study identifies a novel thermoregulatory mechanism in rice: TOGR3 partners with 26S proteasome subunits, including TT1, to drive thermoresponsive ubiquitin–proteasome activity, maintaining sugar homeostasis in stomatal regulation to balance growth and stress resistance. The togr3 mutant shows limited capacity under combined heat–drought stress. TOGR3 also facilitates proteasome self‐recycling to reinforce proteostasis, further enhancing stress adaptation.

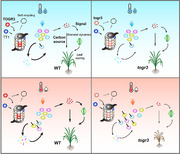

## 1 Introduction

Plant responses to elevated temperatures are primarily mediated by two complementary strategies: thermal adaptive growth and thermotolerance [1, 2, 3, 4, 5, 6]. Plants have evolved sophisticated morphological and physiological adaptations that ensure acclimation to prolonged high‐temperature conditions while maintaining normal growth and reproductive cycles. These processes are orchestrated by complex genetic networks integrating reactive oxygen species (ROS) signaling, miRNA regulation, phytohormone networks, and post‐translational modification‐related pathways, which collectively modulate vegetative and reproductive development—including root and shoot growth, pollen and ovule viability, floral organ formation, and grain filling—under high ambient temperatures [2, 3, 4, 5, 6, 7, 8, 9, 10]. In contrast, thermotolerance represents an immediate protective response that preserves cellular integrity during acute heat damage through rapid physiological adjustments, such as maintenance of ion homeostasis, osmotic regulation, induction of heat shock protein, and activation of antioxidant systems [1, 5, 11]. Elucidating the molecular crosstalk and shared regulatory components between the two strategies is the key to achieving an optimal balance between growth and stress resistance and for breeding broadly thermoadaptive crops.

The 26S proteasome, a key component of the ubiquitin‐proteasome system (UPS), mediates the selective degradation of over 80% of cellular proteins in eukaryotes [12]. It is essential for plant growth, development, and responses to biotic and abiotic stresses [13, 14]. Recent studies have revealed that numerous signaling components involved in plant thermal responses are regulated by the UPS [4, 5, 13, 15]. However, the direct role of the proteasome complex in thermotolerance remains only partially understood. In rice, the core α2 subunit TT1 in 26S proteasome complex, interacts with the SUMO conjugating enzyme SCE1 under heat stress, promoting its ubiquitination and degradation to regulate rice thermotolerance [16, 17]. Modulation of *TT1* haplotypes or knockout of *SCE1* significantly enhances rice yield under high‐temperature conditions [16, 17]. These findings highlight the 26S proteasome as a promising yet underexplored target for improving heat tolerance. Given that the proteasome is a multi‐subunit complex [14, 18], the functions of additional proteasome subunits in environmental adaptation and their coordinated regulation remain largely unknown.

Sugar metabolism, a central aspect of carbohydrate metabolism, serves dual roles in plants by providing carbon for growth and acting as a key signaling hub [19, 20]. Tight regulation of sugar homeostasis is essential for plant responses to biotic and abiotic stresses, including pathogen attack, drought, salinity, cold, and heat [21, 22, 23]. Accumulating evidence indicates that enhancing sugar signaling or accumulation in specific tissues can improve thermotolerance [11, 24, 25, 26, 27, 28]. Moreover, sugar metabolism influences stomatal dynamics: sucrose synthesized in mesophyll cells is transported to guard cells, where elevated sucrose levels promote stomatal closure, whereas reduced levels favor opening [29, 30]. While stomatal regulation is well established in drought resistance, its contribution to thermotolerance remains poorly defined, underscoring the need for further investigation into the role of sugar‐mediated stomatal control under heat stress.

Here, we identify THERMOTOLERANT GROWTH REQUIRED 3 (TOGR3) as a key regulator of thermoresponsive growth and thermotolerance. *TOGR3* encodes the β4 subunit of the 26S proteasome and functions in concert with *TT1* (α2 subunit) to regulate sugar homeostasis, thereby coordinating leaf cooling capacity and adaptive growth under heat stress. Our findings reveal a previously unrecognized proteasome‐based regulatory module that balances plant growth and thermal resilience and provide valuable genetic resources for breeding climate‐resilient crops.

## 2 Results

### 2.1 The *togr3* Mutant Exhibits Impaired Thermal Adaptive Growth and Heat Tolerance

**FIGURE 1:**
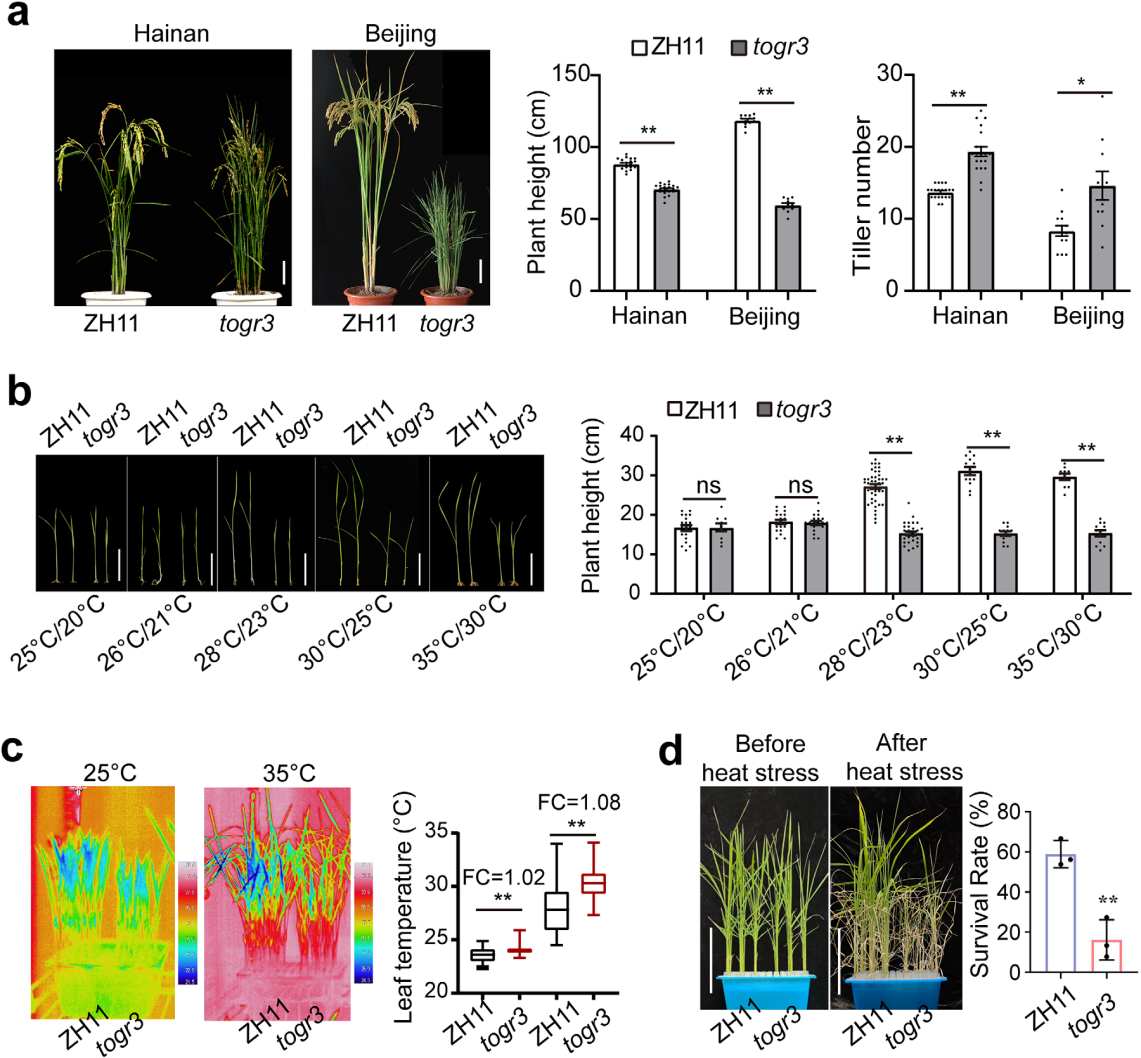
The rice mutant *togr3* exhibits defective thermoadaptive growth and reduced heat tolerance. a) Environment‐dependent phenotypes of *togr3*. Plant height and tiller number under natural field conditions in Hainan during winter and Beijing during summer (*n* ≥ 15). b) Impaired thermoresponsive growth of *togr3* seedlings. Two‐week‐old plants grown in controlled chambers at indicated temperatures with *n* ≥ 20. c) Defective Leaf thermoregulation in *togr3*. Representative thermal images (left) and corresponding boxplots (right) show leaf surface temperatures of seedlings exposed to different growth temperatures. In boxplots, the central line, box, and whiskers of boxplots represent the median, interquartile range (IQR), and 1.5 times the IQR, respectively. FC indicates fold change and *n* ≥ 200 randomly sampled points per genotype were analyzed. d) Reduced heat‐stress tolerance of *togr3*. Seedlings pre‐grown at 25/20°C (day/night) for 2 weeks were subjected to 45°C for 32 h, followed by a 2‐week recovery. Survival rates were analyzed (*n* ≥ 24 and 3 biological replicates). Scale bars: 10 cm (a, b, d). Data are presented as mean ± SEM (a, b, and d) or SD (c), assessed by two‐sided Student's *t*‐test (*p < 0.05; **p < 0.01). All experiments were repeated at least three times with similar results.

EMS mutagenesis of the rice cultivar Zhonghua 11 (ZH11) generated a recessive mutant, *thermotolerant growth required3* (*togr3*), which exhibits temperature‐dependent growth defects. When grown under moderate (Hainan, winter) or elevated (Beijing, summer) temperatures, *togr3* plants displayed pleiotropic developmental abnormalities, including dwarfism, excessive tillering, narrow and curly leaves, reduced seed‐setting rate and hundred‐grain weight, and altered grain morphology, with pronounced environmental plasticity (Figure 1a and Figure S1a–c).

To identify the environmental factors underlying the mutant phenotype, we conducted controlled growth‐chamber experiments. Wildtype ZH11 plants exhibited typical thermomorphogenic responses, characterized by progressive shoot elongation as temperatures increased until reaching heat stress thresholds (Figure 1b). In contrast, *togr3* mutants showed temperature‐dependent growth inhibition: they maintained near‐normal stature at 25°C~26°C but developed severe dwarfism at 28°C~35°C, indicating impaired thermoresponsive growth regulation (Figure 1b). This response was cumulative, with prolonged heat exposure further exacerbating phenotypic severity (Figure S1d,e). In contrast, growth‐chamber experiments manipulating photoperiod revealed no obvious phenotypic differences between wildtype and *togr3* plants, suggesting that photoperiod is not a major environmental determinant of the *togr3* phenotype (Figure S1f). Anatomical analyses further revealed proportional shortening of the first four internodes and complete suppression of the fifth internode in *togr3*, resulting from reduced cell elongation and cell proliferation (Figure S2a,b).

Infrared thermography showed consistently elevated leaf temperatures in *togr3* plants relative to the wildtype under all tested conditions, indicative of impaired thermal regulation (Figure 1c). Consistently, acute heat‐stress assays in juvenile seedlings demonstrated markedly reduced thermotolerance in *togr3*, manifested by rapid desiccation during heat treatment and significantly lower survival rates (Figure 1d and Figure S2c).

These results demonstrate that the *togr3* mutation compromises both thermal adaptive growth and thermotolerance—the two core components of plant thermal adaptation.

### 2.2 *TOGR3* Encodes the Sole β4 Subunit of the Rice 26S Proteasome

**FIGURE 2:**
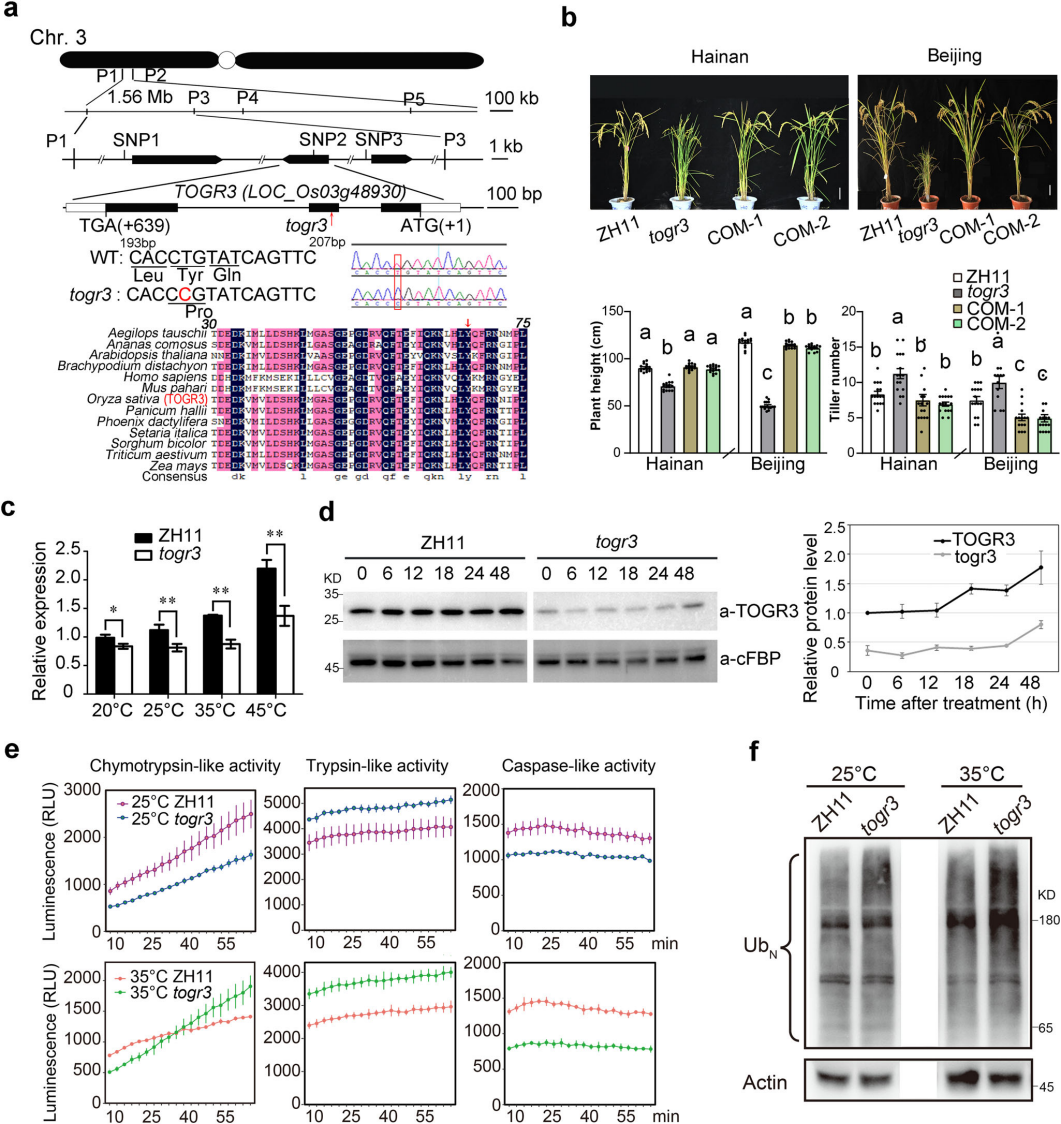
*TOGR3* encodes the β4 subunit of 26S proteasome. a) Identification of *TOGR3*. Top: Fine‐mapping of *TOGR3* on rice chromosome 3. Bottom: multiple sequence alignment of the conserved proteasome β4 subunit across species, highlighting the Leu66Pro mutation in *togr3* (red arrows). b) Genetic complementation of *togr3*. Transformation with the native *TOGR3* genomic fragment rescued plant height and tiller number (*n* ≥ 15). Data: mean ± SEM (Duncan's Multiple Range Test, *p* < 0.05). Scale: 10 cm. COM, complementation lines. c) Thermoresponsive expression of *TOGR3*. RT‐qPCR analysis of *TOGR3* transcripts in seedlings after 2 h temperature treatments. d) Temperature‐dependent accumulation of TOGR3 protein. Immunoblot analysis of wildtype and *togr3* seedlings grown at 25°C and shifted to 35°C for the indicated durations. cFBP served as loading control. e) Proteasome activity assays. Chymotrypsin‐like, trypsin‐like, and caspase‐like activities in wildtype and *togr3* seedlings grown at either 25°C or 35°C for 2 weeks, measured using aminoluciferin‐conjugated substrates (Suc‐LLVY, Z‐LRR, and Z‐LRR, respectively). Activities were assayed at the corresponding growth temperatures. Data represent mean ± SD (*n* = 3). f) Accumulation of ubiquitinated proteins under elevated temperature. Total protein extracts from seedlings grown at 25°C or 35°C were immunoblotted with Ubiquitin antibody. a‐ACTIN served as loading control. All experiments were repeated at least three times.

To identify the causal gene underlying the *togr3* phenotype, we generated an F_2_ mapping population by crossing *togr3* mutant (ZH11 background) with the *Xian*/*indica* variety Nanjing 6. Genetic mapping initially localized the mutation to a 1.56‐Mb region on chromosome 3, which was subsequently narrowed to a 455‐kb interval between markers P1 and P3 (Figure 2a). Whole‐genome resequencing of the mutant revealed three single‐nucleotide polymorphisms (SNPs) within this interval: two located in promoter regions of unrelated genes and one (SNP2) within the second exon of *LOC_Os03g48930*, which encodes the β4 subunit of the 26S proteasome. In the *togr3* mutant, this T‐to‐C substitution results in a Leu66Pro amino acid change within the highly conserved N‐terminal domain of the β4 subunit.

For functional complementation, a 5.5‐Kb genomic fragment encompassing the native promoter (2 kb), the full coding region, and the 3′ untranslated region (1.5 kb) was introduced into *togr3* plants, which fully rescued all mutant phenotypes (Figure 2b). In addition, constitutive overexpression driven by the 35S promoter restored normal growth in the mutant (Figure S3a), whereas RNA interference (RNAi)‐mediated knockdown phenocopied the temperature‐dependent dwarfism in *togr3* (Figure S3b,c). Together, these results confirm *LOC_Os03g48930* as the causal gene underlying the *TOGR3* locus.

Phylogenetic analysis revealed that the proteasome β4 subunit is highly conserved across eukaryotes, existing as a single‐copy ortholog with invariant Leu66 residue from plants to mammals (Figure 2a and Figure S4a), underscoring its functional importance. *TOGR3* was highly expressed in seedling leaves, and the encoded protein was localized to both cytoplasm and nucleus (Figure S4b–d). Structural studies suggest that during β ring–β ring docking in proteasome assembly, two β4 subunits can be positioned in close proximity, raising the possibility of direct β4‐β4 homomeric interaction [31, 32, 33, 34]. We experimentally verified this hypothesis using a luciferase complementation assay and found that the Leu66Pro substitution in TOGR3 markedly weakened this interaction; notably, the mutant togr3 protein () failed to dimerize with the wildtype TOGR3 (Figure S4e). Moreover, both the transcript and protein levels of wildtype *TOGR3* were upregulated under elevated temperatures, while the mutant *togr3* exhibited significantly reduced expression (Figure 2c,d). Together, these findings indicate functional impairment of 26S proteasome in *togr3* plants.

To further assess the functional consequences in *togr3*, we systematically analyzed 26S proteasome activity using fluorogenic peptide substrates. The *togr3* mutant exhibited significantly temperature‐dependent alterations in proteasome activity: chymotrypsin‐like activity was reduced at 25°C and transiently suppressed followed by enhancement at 35°C; trypsin‐like activity consistently was elevated at both temperatures; and caspase‐like activity was severely impaired, particularly at 35°C (Figure 2e). These enzymatic defects correlated with its thermosensitive growth phenotype (Figure 1a,b) and with a marked accumulation of ubiquitinated proteins under high‐temperature conditions (Figure 2f). Collectively, these results demonstrate that the Leu66Pro substitution in the β4 subunit destabilizes proteasome function—especially under thermal stress—thereby compromising protein homeostasis. Our findings establish *TOGR3* as a critical determinant of proteasome activity during high‐temperature adaptation in rice.

### 2.3 Thermoregulatory Function of *TOGR3* via Stomatal Dynamics

**FIGURE 3:**
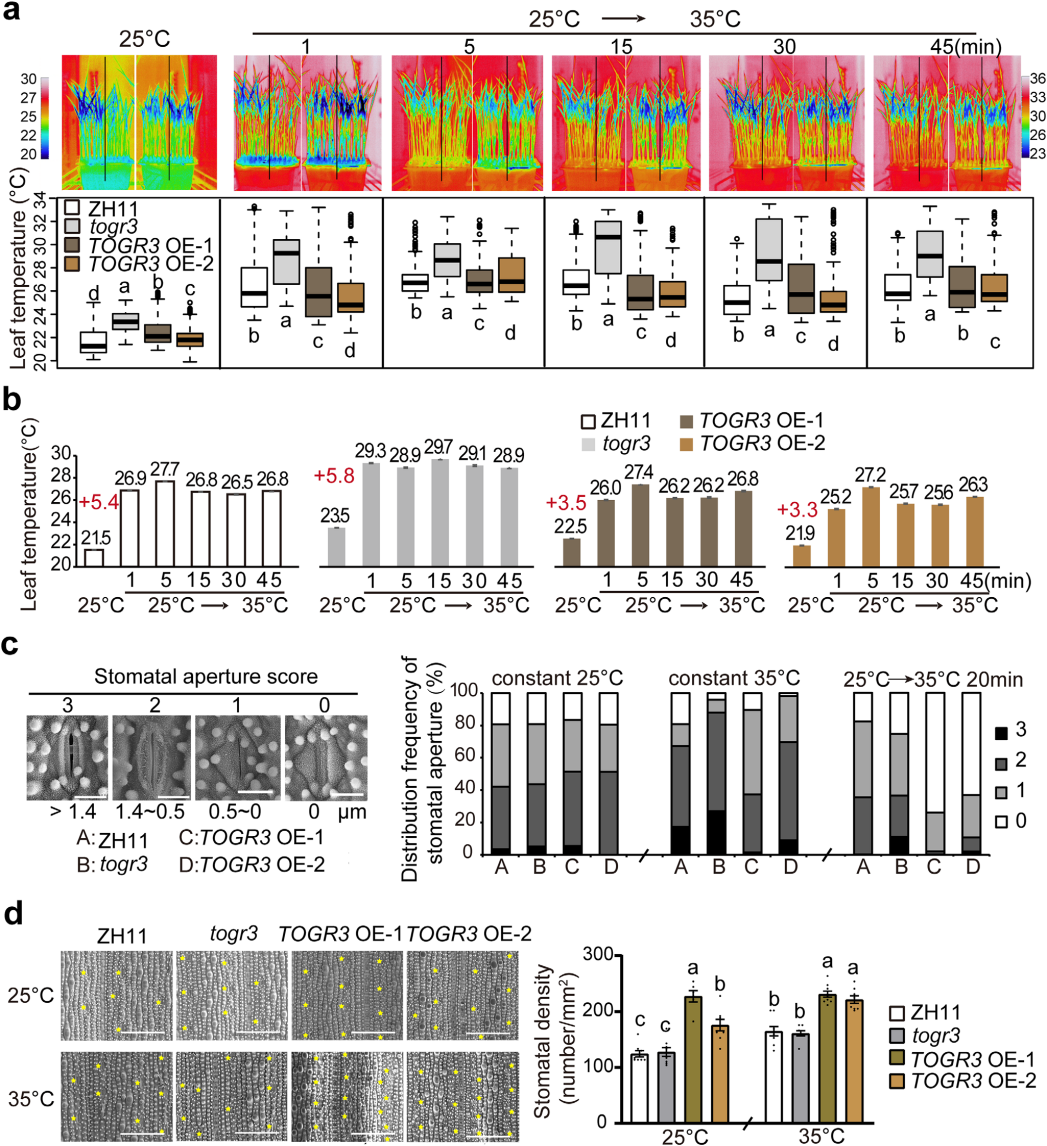
*TOGR3* mediates leaf thermoregulation by modulating stomatal dynamics. a) *TOGR3* suppresses rapid leaf temperature elevation during thermal transition. Top: Representative infrared thermography of wildtype (ZH11), *togr3*, and *TOGR3* overexpression lines before and after a 25°C to 35°C shift. Bottom: Quantitative distribution of leaf temperature. In boxplots, the central line, box, and whiskers represent the median, IQR and 1.5 times the IQR, respectively. *n* ≥ 200 random dots per genotype were analyzed. b) Time‐course analysis of leaf temperature changes corresponding to (a). Red values with plus indicate temperature increase (ΔT) at 1 min after temperature shift. c) *TOGR3* enhances stomatal responsiveness to temperature changes. Cryo‐SEM images and quantitative analysis of stomatal aperture class in seedlings grown at constant temperatures or after 25°C to 35°C shift (20 min). Histograms show distribution frequencies across four aperture classes (fully closed to fully open). N ≥ 120 from 3 to 4 plants. d) *TOGR3* increases stomatal density. Representative images and quantification of stomatal density (cells mm^−2^). Eight images from four plants were measured per genotype. Scale bars: 10 µm (c), 100 µm (d). Data represent mean ± SD (a) or SEM (b, d), and significance in (d) was assessed by Duncan's multiple range test (*p* < 0.05). Experiments were repeated at least twice.

Infrared thermography revealed distinct leaf temperature kinetics among genotypes during transitions from 25°C to 35°C. In wildtype (ZH11) plants, leaf temperature initially measured 21.5°C and exhibited a pronounced fluctuation (ΔT = 5.4°C) within the first minute, peaking at 27.7°C before gradually stabilizing (Figure 3a,b). In contrast, *togr3* mutants showed a higher initial temperature (23.5°C), a greater fluctuation amplitude (ΔT = 5.8°C), and a higher peak temperature (29.7°C). Conversely, *TOGR3*‐overexpressing (OE) lines displayed enhanced thermal buffering capacity, maintaining smaller first‐minute fluctuations (ΔT = 3.3°C–3.5°C) and lower peak temperatures, despite exhibiting slightly higher initial temperatures than the wildtype (Figure 3a,b). These results establish TOGR3 as a critical modulator of leaf thermoregulation that attenuates excessive temperature elevation during thermal fluctuations, thereby potentially mitigating heat‐induced damage.

To elucidate the cellular basis of *TOGR3*‐mediated leaf thermoregulation, we performed cryo‐scanning electron microscopy (cryo‐SEM) analyses of seedling leaves under different temperature regimes. At a constant 25°C, wildtype, *togr3*, and *TOGR3* OE lines exhibited comparable stomatal apertures (Figure 3c). However, during the 25°C to 35°C temperature transition, *togr3* mutants displayed significantly higher proportion of stomata with wider apertures than the wild type and maintained this open state during prolonged exposure to 35°C. In contrast, *TOGR3* OE lines exhibited accelerated stomatal closure immediately following temperature elevation, although these differences diminished under sustained high‐temperature conditions (Figure 3c). This rapid stomatal response likely functions as a thermoregulatory mechanism by limiting hot air influx and helping maintain leaf temperature homeostasis. In addition, *TOGR3* overexpression increased stomatal density under chronic high‐temperature conditions, while exerting minimal effects on other stomatal morphological traits (Figure 3d and Figure S5). Together, these findings demonstrate that *TOGR3* coordinately regulates both stomatal aperture dynamics and stomatal development to optimize leaf cooling capacity under hot conditions.

### 2.4 *TOGR3* Balances Heat and Drought Tolerance with Growth

**FIGURE 4:**
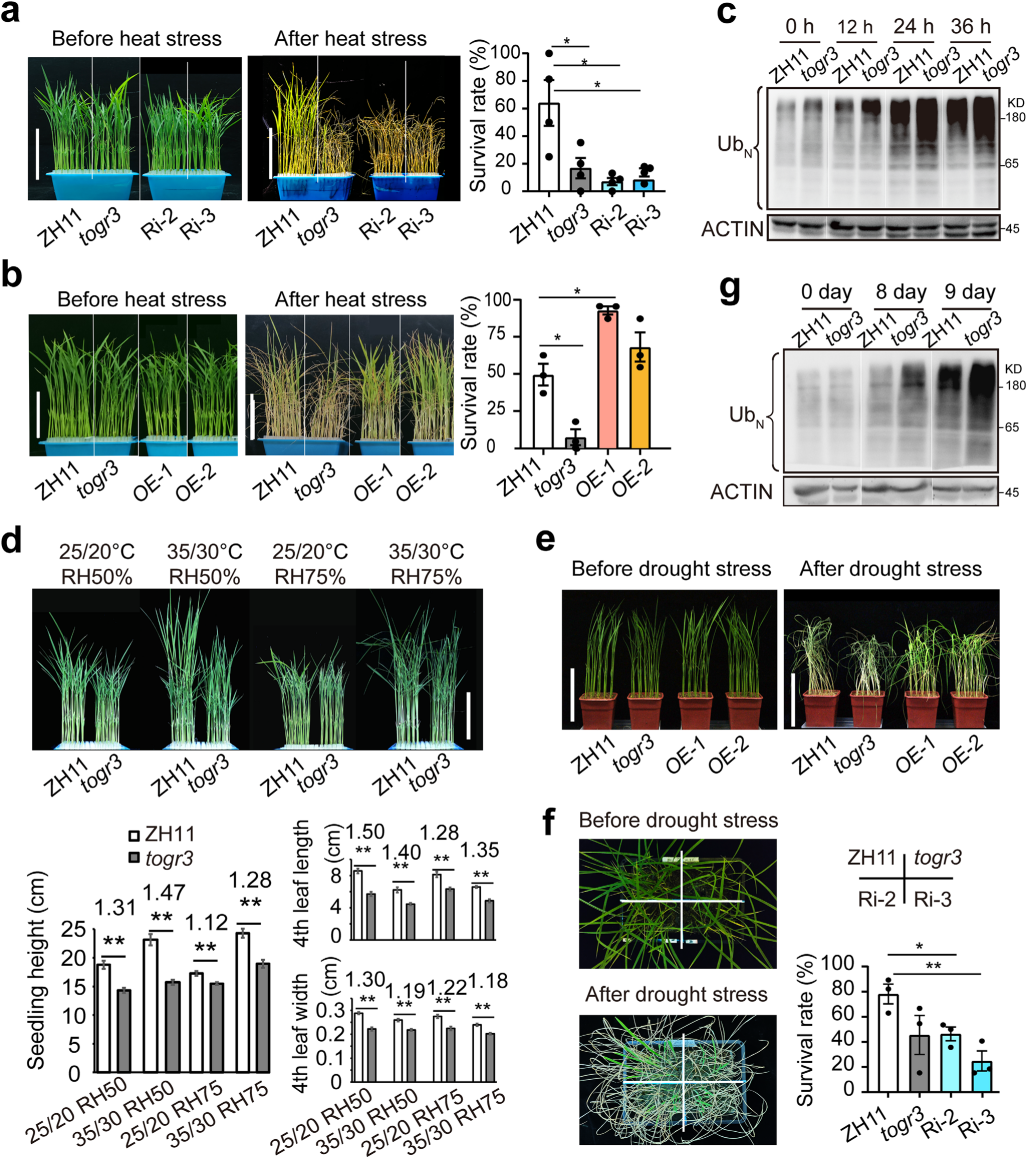
*TOGR3* improves heat and drought tolerance in rice. a,b) Heat tolerance assays. Two‐week‐old seedlings were exposed to 45°C for 32 h (a) or 36 h (b) and recovered for 10 days before survival assessment (*n* ≥ 24, 3–4 biological replicates). Ri, RNAi line; OE, overexpression line. c) Heat‐induced accumulation of ubiquitin conjugates after heat treatment for the indicated durations detected by immunoblotting. a‐ACTIN serves as a loading control. d) Growth responses under combined temperature and humidity conditions. Seedling height and fourth‐leaf dimensions are shown (*n* ≥ 16). Fold changes between wildtype and *togr3* are indicated. e,f) Drought tolerance assays. 2‐week‐old seedlings were subjected to drought for 10 (e) or 12 days (f), followed by 7 days of rewatering (*n* ≥ 24 and 3 replicates). g) Drought‐induced ubiquitin accumulation. Drought treatments for the indicated durations were indicated. Scale bars: 10 cm in (a, b, d, e). Data represent as mean ± SEM and were analyzed using two‐sided Student's *t*‐test (*p < 0.05, **p < 0.01).

To investigate the role of *TOGR3* in thermotolerance, we subjected *TOGR3* RNAi and OE transgenic plants to acute heat stress (45°C). RNAi plants exhibited severely compromised thermotolerance comparable to that of the *togr3* mutant (Figure 4a), whereas OE lines showed markedly enhanced heat resistance (Figure 4b). Molecular characterization revealed accelerated accumulation of ubiquitinated proteins in *togr3* mutants under heat stress compared to wildtype plants (Figure 4c), indicating that disruption of proteostasis underlies the thermosensitive phenotype.

Initial observations of heat‐stressed *togr3* mutants revealed pronounced leaf curling, rapid dehydration, and impaired stomatal regulation (Figure 3c,d; Figures S1c and S2c), suggesting a potential role for *TOGR3* in drought response. Consistently, growth analyses under controlled high‐temperature and low‐humidity conditions showed that *togr3* seedlings developed exacerbated growth defects, including reduced plant height and suppressed leaf expansion (Figure 4d). Drought tolerance assays further demonstrated significantly reduced survival rates in *togr3* and RNAi lines (Figure 4f), whereas OE plants maintained superior water retention and recovery capacity following rewatering (Figure 4e). Notably, drought‐stressed *togr3* mutants accumulated ubiquitinated proteins in a manner similar to that observed under heat‐stress, suggesting that *TOGR3*‐mediated proteostatic regulation is conserved across distinct abiotic stress conditions. Together, these results establish *TOGR3* as a central regulator of stress adaptation that maintains proteostasis to confer both thermotolerance and drought resistance.

**FIGURE 5:**
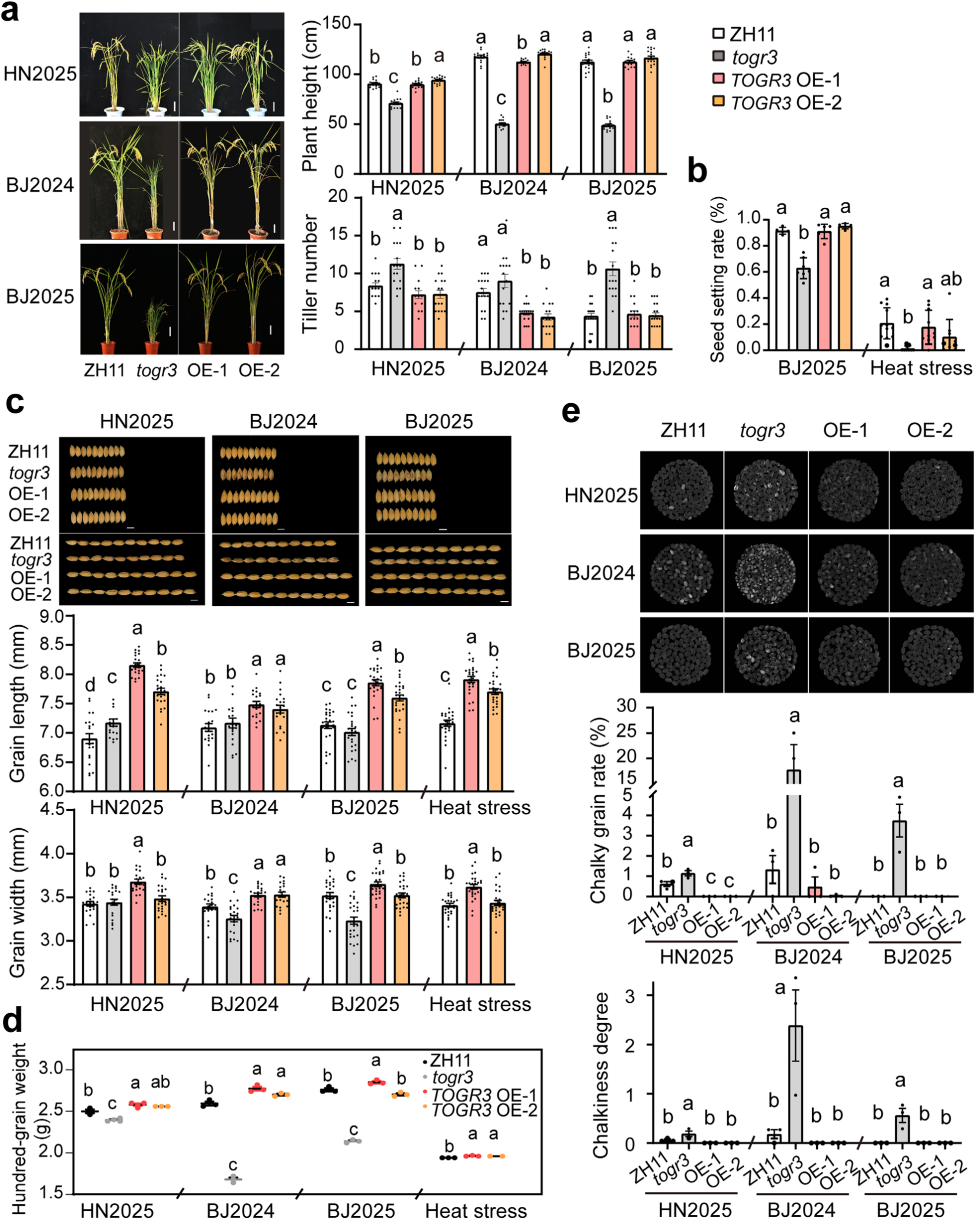
*TOGR3* improves rice grain yield and quality under diverse growth conditions. Agronomic traits of ZH11, *togr3*, and two *TOGR3* overexpression lines were evaluated, including plant architecture (a, *n* ≥ 15), seed‐setting rate (b, *n* ≥ 5), grain size (c, *n* ≥ 20), hundred‐grain weight (d, *n* ≥ 3), and grain quality (e, *n* = 3). Plants were grown under natural field conditions in Hainan winter (HN2025), Beijing summer (BJ2024 and BJ2025), and under heat stress. Scale bars: 10 cm (a), and 5 mm (c). Data: mean ± SEM; analyzed by Duncan's multiple range test (*p* < 0.05).

To evaluate potential growth trade‐offs associated with enhanced stress tolerance coferred by *TOGR3*, we analyzed agronomic traits of wildtype ZH11, *togr3*, and two *TOGR3*‐OE lines grown under natural field conditions across multiple planting seasons, as well as under heat stress in a controlled glasshouse environment. Compared with the wildtype, *togr3* mutant exhibited severe developmental defects, including dwarf stature, excessive tillering, reduced seed‐setting rates, decreased grain size and weight, and compromised grain quality (Figure 5). In contrast, *TOGR3* overexpression did not markedly affect overall plant architecture or seed‐setting rates (Figure 5a,b). Notably, both OE plants displayed increased grain size and hundred‐grain weight under both field conditions and heat stress (Figure 5c,d). Moreover, *TOGR3*‐OE plants consistently produced high‐quality grains with reduced chalkiness compared with both wildtype and *togr3* plants across multiple growing seasons (Figure 5e). Collectively, these findings indicate that *TOGR3* overexpression sustains rice yield and grain quality under both moderate and elevated temperature conditions.

In summary, our results demonstrate that *TOGR3* effectively balances plant growth with resistance to multiple abiotic stresses, highlighting its potential utility for improving crop resilience without yield penalties.

### 2.5 *TOGR3* Mediates a Thermoresponsive Ubiquitylome via Ubiquitin‐Proteasome System

To elucidate how *TOGR3* modulates the ubiquitin‐proteasome system (UPS) during thermal adaptation, we performed a comparative ubiquitinomic analysis of wildtype and *togr3* seedlings grown under moderate (25/20°C) and high (35/30°C) temperature conditions (Tables S1 and S2). Integrated proteome‐ubiquitylome analysis enabled stoichiometric quantification of ubiquitination levels by normalizing ubiquitinated peptides against total protein abundance (Figure S6a). In total, we identified 13193 ubiquitinated peptides corresponding to 6889 protein isoforms, thereby establishing a comprehensive thermoresponsive ubiquitination atlas.

**FIGURE 6:**
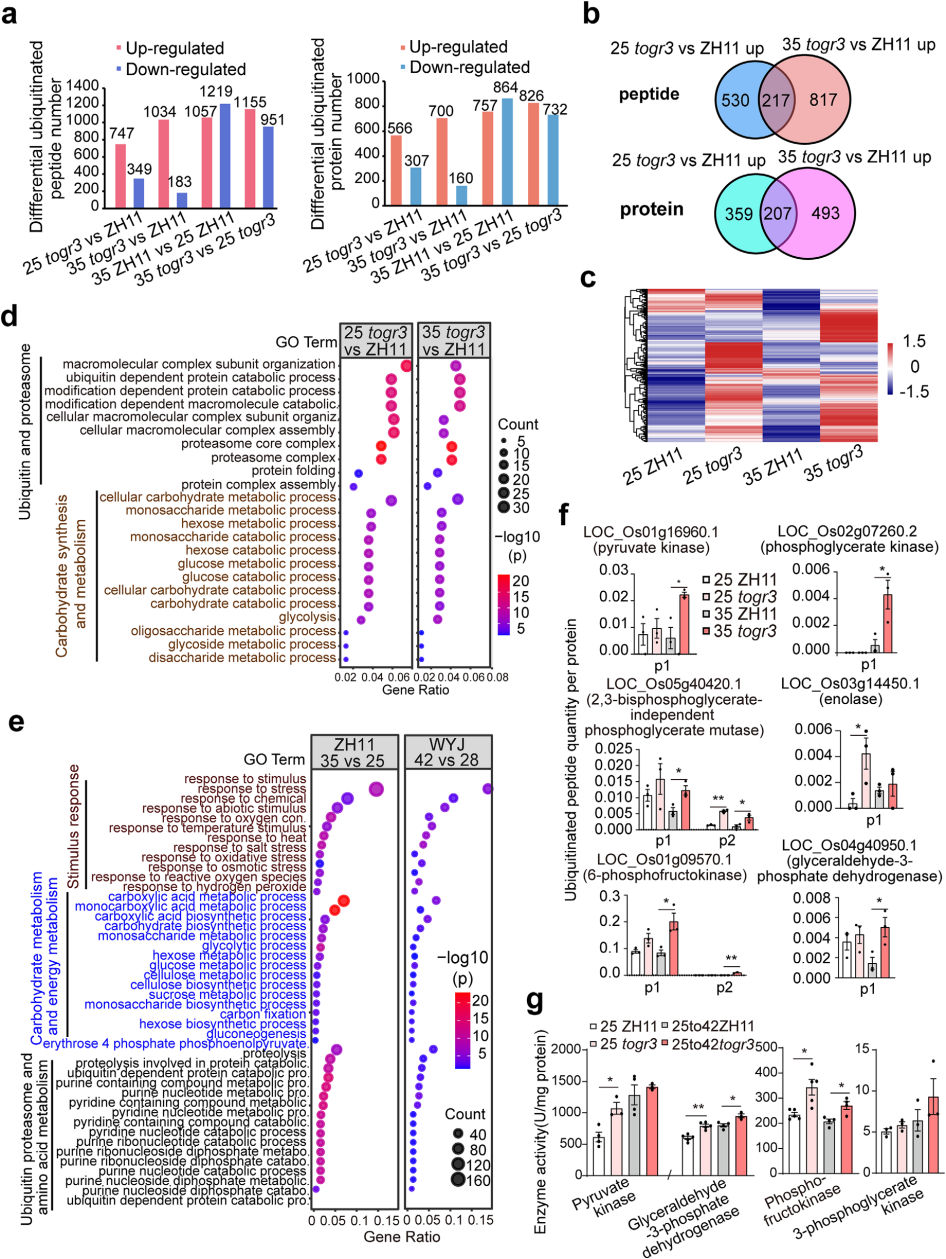
*TOGR3* shapes a thermoresponsive ubiquitylome via ubiquitin‐proteasome system. a) Numbers of differentially ubiquitinated peptides and proteins in ZH11 and *togr3*. Differential ubiquitination was defined as fold change ≥ 2 and Q value < 0.05. b) Comparison of upregulated ubiquitinated peptides and proteins in *togr3 versus* ZH11 between 25°C and 35°C. c) Heatmap showing the relative abundance of upregulated ubiquitinated peptides in *togr3*. d) Shared Gene Ontology (GO) terms among upregulated ubiquitinated proteins at both temperature conditions. e) Overlapping GO biological processes of differentially ubiquitinated proteins between ambient high temperature and acute heat stress datasets in wildtype (WYJ data adapted from Li et al., 2015) [16]. f) Relative abundance of ubiquitinated proteins involved in carbohydrate metabolism. Different Ub‐peptides (P) were labelled. g) Activities of sugar metabolism—related enzymes. Data: mean ± SEM; assessed by two‐sided Student's *t*‐test (*p < 0.05, **p < 0.01).

The *togr3* mutant exhibited pronounced accumulation of polyubiquitinated substrates at both temperatures (747 peptides from 566 proteins at 25°C; 1034 peptides from 700 proteins at 35°C; Figure 6a). The increased ubiquitination burden under elevated temperature correlated with the phenotypic severity and was consistent with western blot evidence of UPS impairment. Although ubiquitinated substrate pools exhibited limited overlap between the two temperature regimes (Figure 6b,c), functional enrichment analyses revealed highly conserved patterns (Figure 6d). Gene Ontology (GO) analysis revealed two major functional categories: (i) UPS autoregulation, including protein folding, ubiquitin‐dependent protein catabolism, and proteasome complex assembly; and (ii) carbohydrate biosynthesis and metabolism, encompassing both mono‐ and polysaccharide pathways (Figure 6d). This convergence suggests that TOGR3 directs UPS activity toward distinct nodal proteins within thermally sensitive regulatory pathways.

Cross‐comparison with a published acute heat‐stress ubiquitylome dataset (Li et al., 2015) [16] convergent in ubiquitinated proteins associated with carbohydrate metabolism, UPS function, and stress response across both chronic warming and acute heat conditions (Figure 6e). Consistently, analysis of the TT1 (proteasome α2 subunit)‐associated ubiquitylome also highlighted carbohydrate metabolism as a prominent category, together with protein/amino acid metabolism and secondary metabolism/defense pathways (Li et al., 2015 [16], Figure S6b). This substantial functional overlap— particularly in carbohydrate metabolism process—supports coordinated action between TOGR3 and TT1 within the proteasome complex.

Notably, numerous enzymes involved in carbohydrate metabolism exhibited elevated ubiquitination in *togr3*, especially under high‐temperature conditions, accompanied by increased protein abundance and enzymatic activity (Figure 6f,g and Figure S6c). Together, these findings reinforce a central role for *TOGR3* in shaping a thermoresponsive ubiquitylome and demonstrate that UPS‐mediated regulation of carbohydrate metabolism is a key mechanism underlying thermal adaptation in rice.

### 2.6 TOGR3‐Dependent UPS Promotes Thermotolerance Through Regulation of Sugar Homeostasis

**FIGURE 7:**
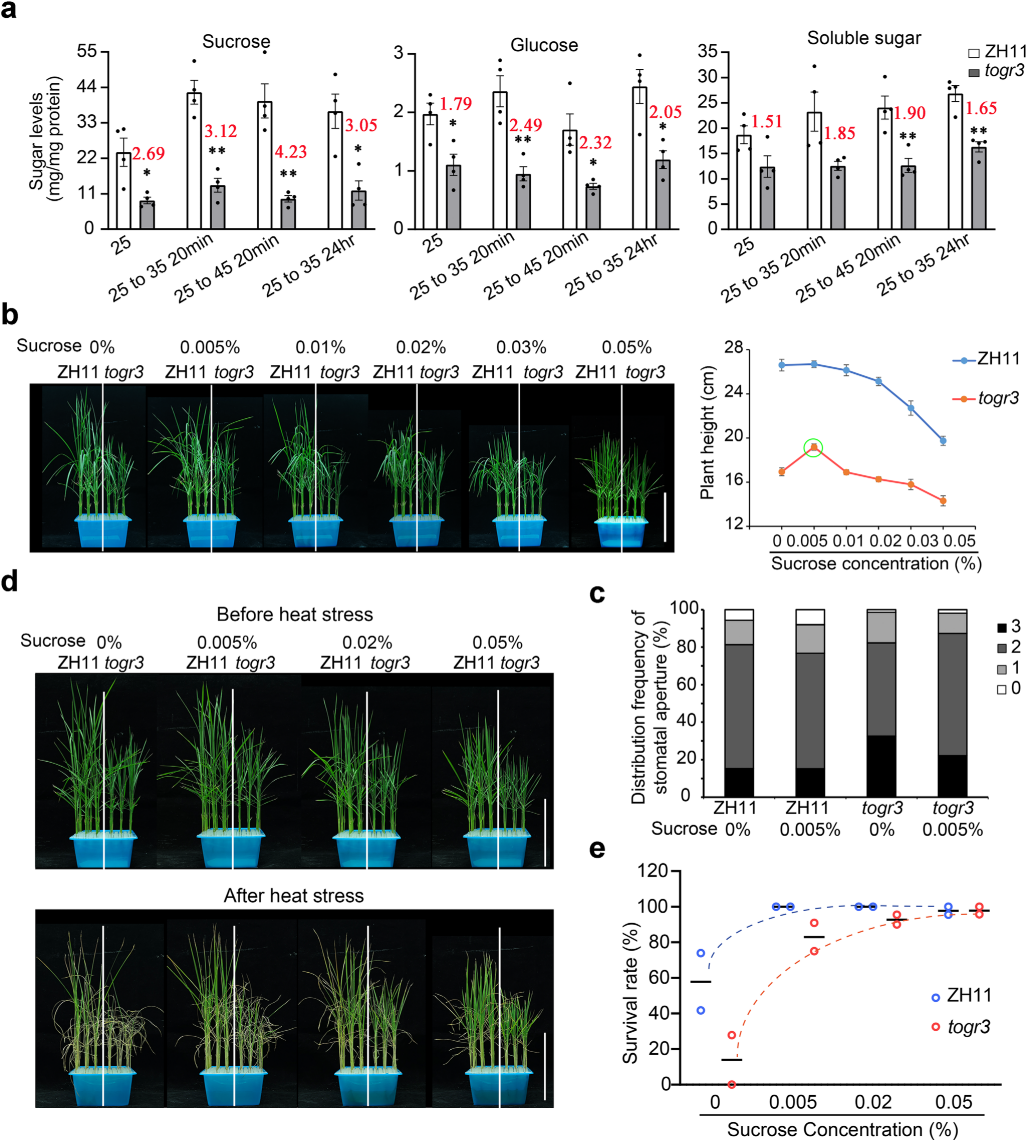
*TOGR3* modulates sugar homeostasis during thermotolerance. a) Dynamics of sucrose, glucose, and total soluble sugar in wildtype and *togr3* seedlings under temperature treatments. Red values indicate fold changes between ZH11 and *togr3*. b) Sucrose‐dependent growth of wildtype and *togr3*. Seedling height was measured after 2‐week growth at 35°C in media supplemented with the indicated sucrose concentrations (%, w/v). Green circle highlights the plant height at 0.005% sucrose. c) Effects of sucrose on stomatal aperture regulation. Stomatal apertures of wildtype and *togr3* seedlings were quantified under 0% or 0.005% sucrose treatment, as described in Figure 3c. d,e) Sucrose enhances thermotolerance. Seedlings pretreated with sucrose (as in b) were exposed to 45°C for 32 h (*n* = 2). Scale bars: 10 cm (b, d). Data: mean ± SEM; assessed by two‐sided Student's *t*‐test (*p < 0.05, **p < 0.01) in (a). Experiments were repeated at least twice.

Ubiquitylome profiling revealed that TOGR3‐dependent UPS activity preferentially targets carbohydrate dynamics, positioning sugar homeostasis as a central downstream output (Figure 6). To directly assess sugar dynamics, we quantified soluble sugars in leaves of wildtype and *togr3* plants. The *togr3* mutant exhibited significantly reduced levels of sucrose, glucose, and total soluble sugars across all tested conditions (Figure 7a). Following temperature elevation, wildtype plants rapidly accumulated leaf sugars, whereas *togr3* showed a blunted response, resulting in progressively widening differences during heat stress (Figure 7a). In contrast, *TOGR3*‐OE lines maintained relatively higher sugar levels (Figure S7a).

Sugar accumulation is tightly linked to stomatal regulation, with elevated sugar levels in guard cells promoting stomatal closure, whereas low levels favor opening [29, 30]. Consistently, TOGR3‐enhanced sugar accumulation correlated with improved stomatal responsiveness and superior leaf thermoregulation capacity (Figure 3), supporting a functional role for sugar homeostasis in thermotolerance via stomatal dynamics.

Exogenous sugar supplementation further substantiated this relationship. Sucrose treatment elicited dose‐dependent effects: wildtype plants exhibited progressive growth inhibition with increasing sucrose concentrations, whereas *togr3* mutants showed partial phenotypic rescue at 0.005% (w/v) sucrose and markedly reduced sensitivity to higher concentrations (Figure 7b). Similar responses were observed with glucose treatment (Figure S7b). The distinct growth responses of *togr3* to elevated sugar levels indicate that exogenous sugars compensate for endogenous sugar deficiency by providing carbon sources, while the attenuated inhibitory effect at high sugar concentrations further suggests that sugar perception and/or signaling pathways are also compromised in the *togr3* mutant.

At the cellular level, 0.005% sucrose treatment partially suppressed excessive stomatal opening in heat‐stressed *togr3* plants relative to sucrose‐free conditions (Figure 7c). Most notably, sucrose pretreatment fully rescued the impaired thermotolerance of *togr3* mutants and further enhanced heat resistance in wildtype plants (Figure 7d,e). Together, these results demonstrate that *TOGR3*‐dependent thermotolerance is mediated through sugar metabolism and signaling, integrating metabolic homeostasis with stress‐adaptive responses.

### 2.7 TOGR3 Regulates Recycling of the 26S Proteasome Complex

Ubiquitylome profiling revealed markedly elevated ubiquitination of most 26S proteasome core particle subunits in the *togr3* mutant at both tested temperatures (Figure S8a). This effect was substantially enhanced at high temperature (35°C), indicating that *TOGR3* is required for proper autoregulatory turnover of ubiquitinated core subunits during thermal adaptation (Figure S8a).

To assess whether this increased ubiquitination affects subunit abundance, we analyzed global proteomic data. Most core particle subunits displayed significantly reduced protein levels in *togr3*, consistent with excessive UPS‐mediated degradation (Figure S8b). These results indicate tightly controlled proteasome subunit turnover is essential for maintaining optimal complex integrity, particularly under environmental stress.

Notably, TOGR3—the sole β4 subunit in rice—exhibited no detectable ubiquitinated peptides in either wildtype or *togr3* plants despite its high protein abundance (Figure S8c). This absence of ubiquitination suggests that TOGR3 functions as an indispensable and non‐redundant regulatory component of the 26S proteasome, playing a central role in proteasome recycling and autoregulation during thermotolerance.

### 2.8 TT1 and TOGR3 Co‐Regulate Rice Thermotolerance

TT1 functions as an α2 subunit of the 26S proteasome and is essential for rice heat tolerance [16]. Proteomic analysis revealed that, similar to TOGR3, TT1 exhibited no detectable ubiquitination while displaying comparable expression patterns (Figure S8c). Although TT1 and TOGR3 do not directly interact within the proteasome complex (Figure S9) [31, 32, 33, 34], Gene Ontology analysis uncovered substantial functional overlap between these two subunits [16] (Figure S6b), suggesting potential synergistic role in thermal adaptation.

**FIGURE 8:**
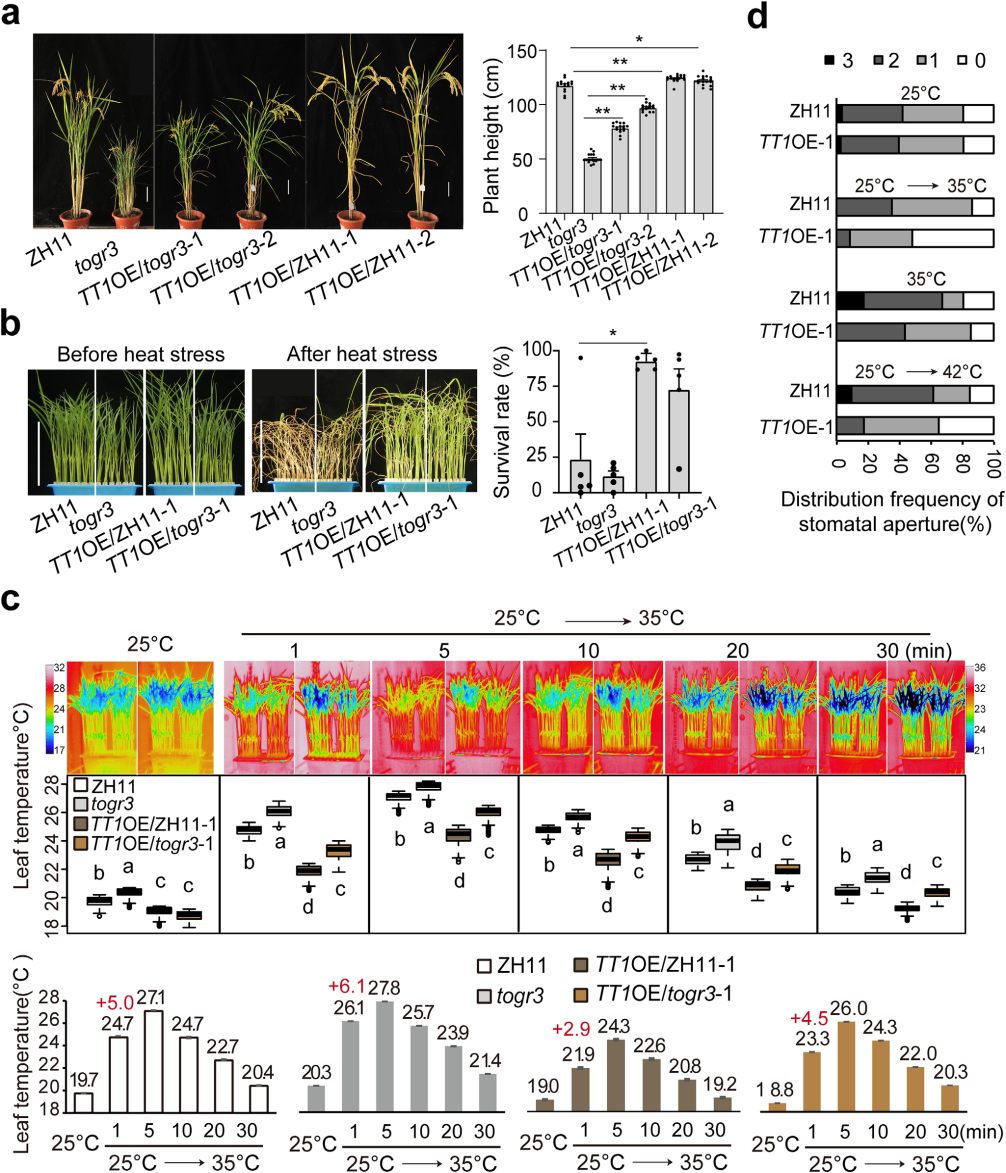
*TT1* partially rescues *togr3's* defects. a) *TT1* overexpression partially rescues the dwarfism of *togr3* (*n* ≥ 15). b) *TT1* enhances thermotolerance in both wildtype and *togr3* backgrounds. Seedlings were exposed to 45°C for 38 h and survival rates were calculated (*n* = 48 and 5 repeats were shown). c) *TT1* improves leaf cooling capacity under heat stress. Infrared thermography (top), temperature quantification (middle), and time‐course analysis (bottom) were performed as described in Figure 3a,b. d) *TT1* accelerates stomatal response to temperature elevation. Methods as Figure 3c. Scale bars: 10 cm in (a, b). Data: mean ± SEM; analyzed by two‐sided Student's *t*‐test in (a, b) or Duncan's multiple range test (*p* < 0.05) in (c).

To test this hypothesis, we generated overexpression lines of *TT1* (derived from the CG14 cultivar [16]) in both wildtype and *togr3* backgrounds. *TT1* overexpression (OE) partially rescued *togr3*’s dwarfism and enhanced plant height in wildtype under elevated temperatures (Figure 8a and Figure S3d). Consistent with previous reports, *TT1* overexpression significantly increased survival rates following heat stress in wildtype backgrounds [16], and substantially restored the compromised thermotolerance of *togr3* mutant (Figure 8b). These findings indicated that TT1 can partially compensate for TOGR3 dysfunction caused by the Leu66Pro mutation.

To determine whether *TT1* and *TOGR3* share a conserved thermoregulatory mechanism, we monitored leaf temperature dynamics in ZH11, *togr3*, and *TT1*‐OE lines under ZH11 and *togr3* backgrounds during temperature transitions. Within the first minute of thermal stimulation, *TT1*‐OE plants exhibited superior thermal buffering capacity (ΔT = 2.9°C, peaking temperature 21.9°C), representing a 2.1°C reduction compared with wildtype, and reduced temperature fluctuation in *togr3* background from 6.1°C to 4.5°C (Figure 8c). Cryo‐scanning electron microscopy further revealed that *TT1* overexpression accelerated stomatal closure following temperature elevation (Figure 8d), phenocopying *TOGR3*‐mediated regulation of stomatal dynamics.

**FIGURE 9:**
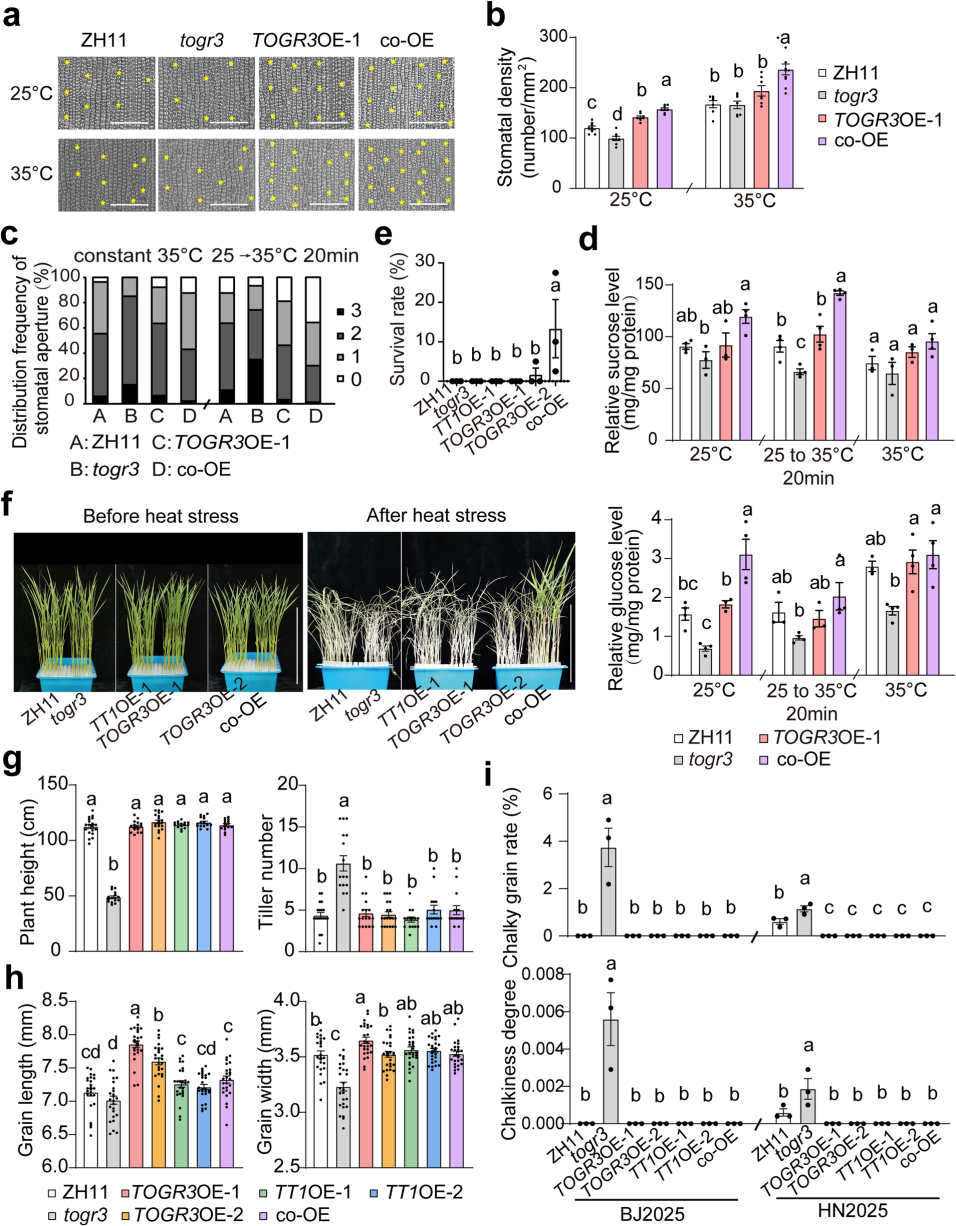
*TOGR3* and *TT1* act synergistically to enhance rice thermotolerance. a,b) Co‐overexpression (co‐OE) of *TOGR3* and *TT1* increases stomatal density (*n* = 8). c) Co‐OE enhances stomatal responsiveness to thermal transitions. Methods as Figure 3c (*n* ≥ 120). d) Increased sucrose and glucose levels in co‐OE plants. e,f) Enhanced thermotolerance conferred by *TOGR3* and *TT1* co‐OE. Seedlings were exposed to 45°C for 50 h and survival rates were assessed after 2 weeks of recovery (*n* = 3). g–i) Plant architecture (g), grain size (h) and grain quality (i) of all genotypes were analyzed under Beijing summer field conditions (2025). Data collection and sample sizes were as described in Figure 5. Scale bars: 100 µm (a) and 10 cm (f). Data represent mean ± SEM, and significance was assessed by Duncan's multiple range test (*p* < 0.05).

To assess the combined effects of these two subunits, we generated a *TOGR3*‐*TT1* co‐overexpression (co‐OE) line by genetic crossing. Compared with *TOGR3* single overexpression, co‐OE plants exhibited increased stomatal density, enhanced stomatal aperture dynamics, and elevated cellular sugar levels (Figure 9a–d). Heat‐stress assays demonstrated that co‐OE seedlings displayed greater thermotolerance than either single overexpression lines (Figure 9f). Agronomic analyses revealed that *TOGR3*–*TT1* co‐OE plants maintained plant architecture and grain quality comparable to single‐subunit OE lines; however, improvements in grain size and hundred‐grain weight were less consistent than those observed in *TOGR3*‐OE plants across certain growing seasons (Figure 9g–i and Figure S10).

Collectively, these results demonstrate that *TOGR3* and *TT1* act cooperatively to enhance rice thermotolerance while maintaining yield stability and grain quality across fluctuating temperature conditions.

## 3 Discussion

**FIGURE 10:**
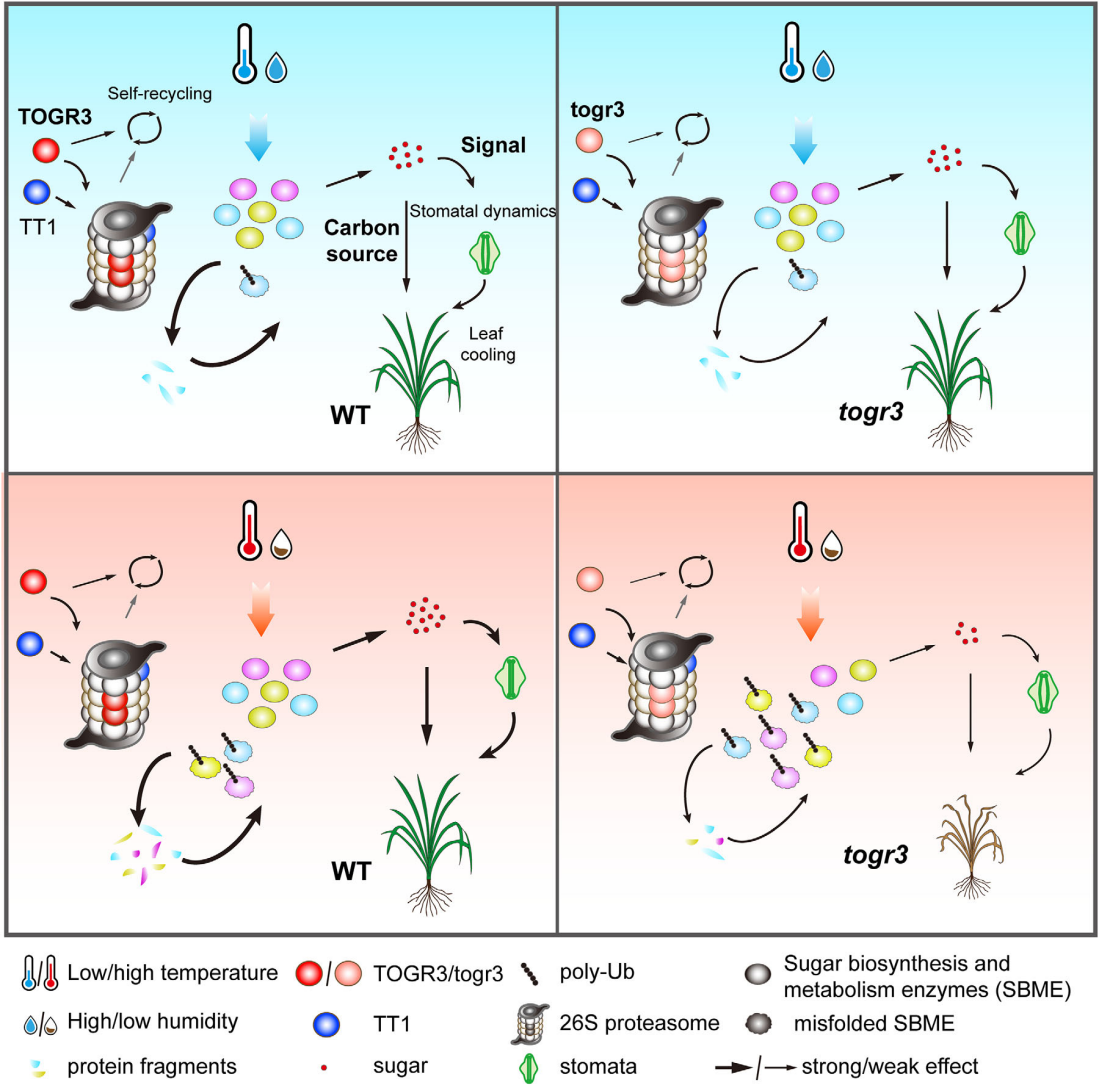
Proposed model illustrating the molecular mechanism by which TOGR3‐dependent ubiquitin‐proteasome system regulates rice thermotolerance through sugar homeostasis.

This study uncovers a previously unrecognized thermoregulatory mechanism in rice. Under moderate conditions, TOGR3 assembles with other 26S proteasome subunits, including TT1, to form a functional ubiquitin–proteasome system (UPS) that mediates thermoresponsive ubiquitylation (Figure 10). This process promotes the recycling of misfolded proteins in sugar biosynthesis and metabolism, thereby sustaining leaf sugar homeostasis required for carbon utilization as well as stomatal dynamic regulation, including aperture control and developmental patterning. These coordinated effects enhance leaf cooling and support plant growth. Although the Leu66Pro TOGR3 variant retains partial UPS activity under non‐stress conditions, combined heat and drought stress exceed its protein‐recycling capacity, leading to reduced sugar accumulation, growth inhibition, and compromised stress tolerance relative to wildtype. In addition, TOGR3 facilitates 26S proteasome self‐recycling, further reinforcing proteostasis under stress. Together, these integrated mechanisms enable robust abiotic stress adaptation across both vegetative and reproductive stages (Figure 10).

### 3.1 The Dual‐Safeguard Thermoregulatory Mechanism Balances Plant Growth and Thermotolerance

Plant responses to elevated environmental temperatures are primarily mediated through two complementary processes: thermoresponsive growth and thermotolerance [1, 2, 3, 4, 5, 6]. Thermoresponsive growth is typically manifested as gradual morphological and developmental changes across different ambient temperatures, reflecting long‐term acclimation. In contrast, thermotolerance involves rapid physiological and biochemical responses that protect cellular integrity and determine survival under acute heat stress. Coordinating these two processes is critical for optimizing the trade‐off between growth and stress defense.

Although most studies have focused on either thermoresponsive growth or thermotolerance in isolation, several regulators—including *TOGR1*, *AET1*, *PIF4*, and *COP1*—have been implicated in both processes [9, 35, 36, 37]. However, whether these dual phenotypes are governed by shared or distinct molecular mechanisms remains largely unresolved. Here, we demonstrate that mutation of *TOGR3* leads to distinct, temperature‐dependent defects in both thermoresponsive growth and thermotolerance (Figures 1 and 4). Importantly, *TOGR3* regulates these phenotypes through a common mechanistic framework, indicating that TOGR3 and its associated proteasome complex function as a critical molecular nexus linking developmental plasticity and stress protection.

This dual‐safeguard mechanism integrates rapid physiological acclimation with longer‐term developmental adjustments, thereby alleviating the classical antagonism between stress tolerance and growth or yield penalties. From an applied perspective, enhancing the expression of key proteasome subunit genes such as *TOGR3* or *TT1*, or deploying superior allelic variants, represents a promising strategy for breeding thermotolerant crops. Nonetheless, how overexpression of individual or multiple proteasome subunits enhances overall proteasome function remains unclear, and whether additional subunits exert comparable effects warrants further investigation. Identifying rate‐limiting components of the proteasome complex and elucidating their coordinated regulation will be essential to fully exploit this pathway for crop improvement under heat stress.

### 3.2 The TOGR3‐Mediated UPS Represents a Potentially Universal Mechanism for Plant Abiotic Resilience

Under natural field conditions, abiotic stresses often occur in combination, with heat and drought representing the most common and agronomically relevant stress pair [38, 39]. Classical studies have suggested that plant responses to heat and drought are governed by largely antagonistic regulatory mechanisms, complicating efforts to engineer crops with simultaneous resistance to both stresses [39, 40]. In contrast, our findings demonstrate that the TOGR3‐mediated ubiquitin–proteasome system (UPS) concurrently enhances thermotolerance and drought resistance (Figure 4), indicating that this module functions as a shared and integrative stress‐resilience mechanism. This role is consistent with the fundamental biological function of the UPS as the primary protein degradation pathway in eukaryotic cells, responsible for the turnover of more than 80% of cellular proteins in plants [14, 18, 41]. Abiotic stresses such as heat and drought induce widespread protein misfolding and damage. To preserve cellular homeostasis, these aberrant proteins are selectively recognized, polyubiquitinated, and degraded by the UPS, while the released amino acids are recycled to support de novo protein synthesis. Accordingly, the UPS has been implicated in nearly all major abiotic stress responses, underscoring its central and indispensable role in plant stress adaptation [13, 18, 42, 43].

The utilization of this common UPS‐dependent mechanism to buffer diverse environmental challenges highlights both its evolutionary conservation and functional versatility. Based on our results, we propose that strengthening TOGR3‐mediated proteasome function represents a promising strategy for broadly enhancing tolerance to multiple abiotic stresses. Future studies will explore the involvement of this module in additional stress‐response pathways and assess its potential to stabilize yield performance under complex, real‐world agricultural conditions.

### 3.3 The UPS‐Dependent Sugar Metabolism and Proteasome Recycling Are Critical for Plant‐Environment Interaction

While the ubiquitin‐proteasome system (UPS) is generally regarded as a broad‐spectrum protein degradation machinery with limited substrate specificity [18, 41, 43], our ubiquitylome analyses unexpectedly revealed a pronounced enrichment of targets involved in carbohydrate/sugar metabolism and proteasome recycling, highlighting their central role in stress resilience. These findings uncover a refined regulatory mechanism in which the UPS selectively modulates the turnover of sugar‐metabolizing enzymes during heat stress, thereby promoting rapid accumulation of soluble sugars in leaf tissues (Figure 6).

This UPS‐driven sugar regulation confers dual protective benefits. In the short term, elevated sucrose levels promote stomatal closure and osmotic adjustment, reducing heat‐induced water loss and cellular damage. In the longer term, sustained sugar availability supports increased stomatal density, enhanced transpiration capacity, and carbon supply for adaptive growth, collectively facilitating thermal acclimation.

Beyond vegetative tissues, our grain trait analyses suggest that this mechanism may also influence carbon and nitrogen allocation during later stages of crop development, particularly under elevated temperatures. Environmental temperature strongly affects the balance between protein and starch accumulation in grains. Notably, Li et al. reported that the β subunit of SEC61, a core component of the endoplasmic reticulum‐associated degradation (ERAD) pathway, mediates high‐temperature‐dependent carbon and nitrogen reallocation in rice and profoundly impacts grain chalkiness [44]. This phenotype closely parallels the effects observed for *TOGR3*, suggesting potential functional convergence or crosstalk between these proteostasis pathways. Elucidating the relationship between UPS‐ and ERAD‐mediated regulation will be critical for understanding how environmental temperature shapes crop growth and quality.

Intriguingly, TOGR3‐dependent UPS activity also enhances proteasome recycling by accelerating the turnover of 26S proteasome subunits under stress (Figure S8). Among the 20S core particle subunits, TOGR3 and TT1 are unique in lacking detectable ubiquitination in rice. While TT1 orthologs in other species exhibit ubiquitination, TOGR3 represents the sole non‐ubiquitinated β4 subunit in rice. This distinctive property may be related to the structural features of the β4 subunit, as human 20S proteasome studies have identified a specialized ligand‐binding interface within β4–β4 homodimers [34]. The molecular basis by which β4 contributes to proteasome self‐recycling and stress adaptation warrants further biochemical and structural investigation.

### 3.4 The UPS Coordinates Sugar Homeostasis to Mediate a Novel Thermoregulatory Mechanism in Plants

Leaf cooling has traditionally been attributed primarily to transpiration [45, 46]. Here, we uncover an additional, previously unrecognized thermoregulatory strategy in plants (Figures 3 and 8). Immediately following heat shock—and prior to the onset of transpiration—plants rapidly induce stomata closure to minimize hot air influx, thereby limiting the initial rise in leaf temperature and reducing thermal damage. Subsequently, stomata gradually reopen to activate transpiration, enabling sustained temperature regulation. These findings reveal a more sophisticated, temporally staged heat‐response strategy than previously appreciated, reflecting an evolutionarily optimized adaptation to transient heat stress. The TOGR3‐mediated UPS coordinates this process through selective protein turnover, representing a conceptual advance in our understanding of plant thermotolerance mechanisms.

In summary, the β4 subunit of the 26S proteasome, TOGR3, cooperates with the α2 subunit TT1 to regulate sugar homeostasis via the UPS, thereby balancing rice growth, development, and stress tolerance. This work provides new mechanistic insights into proteasome‐mediated stress adaptation and identifies valuable genetic resources for breeding climate‐resilient crops. Future studies will further explore the broader roles of the 26S proteasome in crop stress resilience and advance its application in climate‐adaptive breeding strategies.

## 4 Experimental Section

### 4.1 Plant Materials and Growth Conditions

Rice (*Oryza sativa subsp. geng cv*. Zhonghua 11) seeds were mutagenized with ethyl methanesulfonate (EMS). T_2_ plants were screened for dwarf, environment‐sensitive phenotypes in Beijing (summer) and Hainan (winter). Field phenotyping was conducted under representative seasonal conditions: Beijing (March–October) and Hainan (December–April). Seedling phenotyping was conducted in growth chambers (Panasonic MLR‐352H‐PC) using liquid culture under controlled conditions: 16 h light/8 h dark cycle, 50% relative humidity, and 16 000 lx light intensity. Moderate and high temperature regimes were set at 25/20°C and 35/30°C (day/night), respectively.

### 4.2 Map‐Based Cloning of *TOGR3*

To generate a mapping population, the *togr3* mutant (ZH11 background) was crossed with the Xian variety Nanjing 6. F_2_ individuals displaying severe dwarfism under Beijing field conditions were selected for bulked segregant analysis using polymorphic SSR markers. The mutation was initially mapped to a 1.56‐Mb interval on chromosome 3 between markers P1 and P2, and subsequently fine‐mapped to a 455‐kb region between markers P1 and P3. Resequencing of this interval identified three SNPs, of which only SNP2 was located within the coding region of *LOC_Os03g48930*, identifying it as the candidate gene.

### 4.3 Genetic Complementation and Transgenic Analyses

For genetic complementation, a 5.5‐kb genomic fragment of ***TOGR3*** (2.0 kb native promoter, coding sequence, and 1.5 kb 3’ UTR) or a 636‐bp coding sequence (CDS) was amplified and cloned into ***pCAMBIA1300*** *or* ***pBWA(V)*** *HS*, respectively. The construct ***pCAMBIA1300‐pTOGR3::TOGR3*** was introduced into the *togr3* mutant to generate complementation lines. ***pBWA(V)HS‐35S::TOGR3‐3×FLAG*** was transformed into ZH11 and *togr3* to generate overexpression lines, whereas ***pBWA(V)HS‐35S::TOGR3‐RNAi*** was transformed into ZH11 to obtain RNAi lines. The ***TT1*** CDS was amplified from the CG14 variety [16], cloned into *pCAMBIA1300‐pUbiquitin::TT1*, and transformed into ZH11 and *togr3* to generate *TT1* overexpression lines. Primer sequences used in this study are listed in Table S3.

### 4.4 Heat Tolerance Assessment

Seedlings were grown for 2 weeks under control conditions (25/20°C day/night, 16/8 h photoperiod, 50% humidity, 16 000 lx). Heat stress was applied at 45°C for indicated durations without changing other conditions, followed by a 10‐day recovery under control conditions before survival assessment. For agronomic evaluation, plants were grown in a glasshouse with daily maximum temperatures maintained at 30°C–35°C during the seedling stage and 40°C–45°C during flowering and grain filling, representing 5°C–10°C above normal field conditions.

### 4.5 Drought Tolerance Assessment

Two‐week‐old soil‐grown seedlings (35/20°C day/night, 16/8 h photoperiod, 50% humidity, 16 000 lx light intensity) were subjected to water withholding for 10–12 d, followed by 7 d of rewatering before phenotypic evaluation.

### 4.6 Proteasome Activity Assay

Proteasome activities were measured using the Proteasome‐Glo Cell‐Based Assay kit (Promega, #G1180). Total protein was extracted in proteasome buffer (50 mm Tris pH 7.5, 5 mm MgCl_2_, 250 mm sucrose, 2 mm ATP, 1 mm DTT, 0.5 mm EDTA, 5% glycerol) and quantified by Bradford assay. Protein samples (1–5 µg) were incubated with specific luminescent substrates for chymotrypsin‐like, trypsin‐like, and caspase‐like activities at 35°C for 10 min. Luminescence was recorded using a GloMax 20/20 luminometer (Promega, #PAE5311).

### 4.7 Ubiquitin‐Enriched Proteomics

#### 4.7.1 Plant Material and Protein Extraction

Leaves from 2‐week‐old wildtype and *togr3* seedlings grown at 25/20°C and 35/30°C (three replicates per condition). Homogenized rice powder (0.6 g) in 4.5 mL Urea Lysis Buffer (20 mm HEPES pH 8.0, 8 M urea, 1 mm sodium orthovanadate, 2.5 mm sodium pyrophosphate, 1 mm β‐glycerophosphate). Ubiquitin‐modified peptide were enriched by using PTMScan Pilot Ubiquitin Remnant Motif (K‐ε‐GG) Kit (Cell Signaling Technology, #14482) and performed according to Udeshi et al. (2013) [47] with modifications. Synchronous proteomic analysis is used as an internal reference to calculate the percentage of ubiquitination modification for a peptide.

#### 4.7.2 Protein Processing

Quantification: BCA assay (adjusted to 2 µg µL^−1^). Reduction: 1/250 vol 1.25 M DTT (5 mm final), 45 min RT. Alkylation: 95 mg iodoacetamide in 5 mL H_2_O → 1/10 vol (10 mm final), 30 min RT (dark). Urea dilution: +16 mL 20 mm HEPES (< 2 M final). Digestion: Trypsin (1:100 w/w, 80 µg per 8 mg protein), 30°C overnight. Peptide Purification: Acidification: 150 µL 20% TFA, 3000 × g 10 min. Sep‐Pak C18: Activation: 5 mL 100% CAN. Wash: 5 mL 0.1% TFA/50% CAN. Equilibration: 1 + 5 + 6 mL 0.1% TFA. Elution: 3 × 2 mL 0.1% TFA/50% ACN (6 mL total). Lyophilization: Dry eluate, reserve 80 µL for proteomics. Ubiquitin Enrichment: K‐ε‐GG Antibody Beads: Crosslink: 20 mm DMP in 100 mm borate pH 9.0, 30 min RT. Block: 200 mm ethanolamine pH 8.0, 4°C 2 h. Immunoaffinity: Incubate peptides (1.4 mL IAP buffer) with beads, 4°C 2 h. Wash: 3 × 1.4 mL IAP buffer, 2 × 1.2 mL H_2_O. Elute: 2 × 50 µL 0.15% TFA.

#### 4.7.3 LC‐MS/MS Analysis

Instrumentation: Orbitrap Eclipse Tribrid MS (Thermo Scientific) coupled to Easy n‐LC 1200 HPLC. Chromatography: Trap column: 100 µm × 2 cm (Reprosil‐Pur C18 AQ, 5 µm, Dr. Maisch GmbH); Analytical column: 75 µm × 25 cm (Reprosil‐Pur C18 AQ, 1.9 µm, Dr. Maisch GmbH); Gradient: 103‐min linear from 4% to 99% B (A: 0.1% FA/H_2_O; B: 80% ACN/0.1% FA). MS Parameters: DIA mode with 40 variable windows (16 m/z isolation width); MS1: 120 000 resolution (400–1210 m/z); MS2: 30 000 resolution (200–2000 m/z); HCD collision energy: 30%; Ion source: 2.0 kV spray voltage, 320°C capillary temperature.

#### 4.7.4 Data Analysis

Raw DIA files were analyzed using Spectronaut 19.9 (Biognosys) in DirectDIA mode against the Oryza sativa MSU7 database. Key parameters: Enzyme: Trypsin (≤ 2 missed cleavages). Mass tolerances: 10 ppm (precursor), 0.02 Da (product ions). Modifications: Fixed: Carbamidomethylation (C); Variable: Oxidation (M), GlyGly (K). FDR control: <1% at peptide/protein levels. Normalization: Cross‐run calibration using proteome data as reference. GO enrichments: agriGO v2.0 [48].

### 4.8 Leaf Temperature Measurement

Thermal images were captured using an infrared camera (Avio R500) following transfer of seedlings from 25°C to 35°C. Leaf temperatures were quantified using InfReC Analyzer software. At least 200 data points from multiple plants were used for statistical analysis.

### 4.9 Stomatal Analysis

Lower epidermal cells from the fourth leaf were examined by Cryo‐SEM (HITACHI S‐3000N with Quorum PP3000T). Seedlings were grown at 25/20°C or 35/30°C, with some plants shifted to 35°C or 42°C for 20 min prior to sampling. Stomatal density, aperture, and morphology were quantified from 4 to 5 biological replicates.

### 4.10 Exogenous Sugar Treatment and Endogenous Sugar Measurement

Wildtype and *togr3* seedlings were cultured in lipid‐supplemented media containing graded concentrations of sucrose or glucose (0%–0.05% w/v) under high ambient temperature (35°C/30°C) 3 days post germination. Following a 2‐week acclimation period in sugar‐supplemented media, plants were subjected to phenotyping or heat stress treatment (42°C for 32 h). The levels of sucrose, glucose, and total soluble sugars in the 4th leaves of 2‐week‐old seedlings were measured using commercial kits (Boxbio, Beijing) before and after heat treatment, and normalized to the total soluble protein content.

### 4.11 Enzyme Activity Assays

Activities of pyruvate kinase, phosphofructokinase, glyceraldehyde‐3‐phosphate dehydrogenase, and phosphoglycerate kinase were measured in fourth‐leaf extracts using commercial kits (Boxbio, Beijing) before (25°C) and after heat stress (45°C, 20 min). Activities were normalized to total soluble protein.

### 4.12 Luciferase Complementation Imaging

Coding sequences were cloned into nLUC or cLUC vectors and transiently expressed in *Nicotiana benthamiana* via Agrobacterium infiltration. Luminescence was detected 36 h post‐infiltration after luciferin application using a Berthold LB985 imaging system. (Berthold, Germany).

### 4.13 Statistical Analysis

Data are presented as means ± SEM. Statistical significance between two groups was assessed using a two‐sided Student's *t*‐test (*p* < 0.05, *p < 0.01), while multiple comparisons were performed using Duncan's multiple range test (*p* < 0.05) in RStudio. Source data are provided with this paper (Table S4 and S5). Stress assays were repeated three to five times, and all other experiments were performed at least twice.

## Author Contributions

B.Z., X.W., T.X., and F.G. contributed equally to this work. Y.X. conceived and designed the project. B.Z., X.W., T.X., and F.G. performed the main experiments and analyzed the main data. B.Z., X.W., and F.G. interpreted the data. B.Z. wrote and Y.X. revised the manuscript. X.S., C.M., Y.W., X.H., and H.Z. assisted in the planting, genotyping, seed harvest, and genetic crossing.

## Conflicts of Interest

The authors declare no conflicts of interest.

## Supporting information




**Supporting file 1**: advs73909‐sup‐0001‐SuppMat.docx

**Supporting file 2**: advs73909‐sup‐0002‐TableS1.xlsx

**Supporting file 3**: advs73909‐sup‐0003‐TableS2.xlsx

**Supporting file 4**: advs73909‐sup‐0004‐TableS3.xlsx

**Supporting file 5**: advs73909‐sup‐0005‐TableS4.xlsx

**Supporting file 6**: advs73909‐sup‐0006‐TableS5.xlsx

## Data Availability

The data that support the findings of this study are available in the supplementary material of this article. Source data of the main and supplemental figures are in Table S4 and S5. The raw data of ubiquitylome and proteome presented herein have been deposited in OMIX, China National Center for Bioinformation/Beijing Institute of Genomics, Chinese Academy of Sciences, under the accession no. OMIX010773. Interested readers can access the data repository at https://ngdc.cncb.ac.cn/omix [49, 50].

## References

[1] Y. Kan , X. R. Mu , J. Gao , H. X. Lin , and Y. Lin , “The Molecular Basis of Heat Stress Responses in Plants,” Molecular Plant 16 (2023): 1612–1634. 37740489 10.1016/j.molp.2023.09.013

[2] J. J. Casal and S. Balasubramanian , “Thermomorphogenesis,” Annual Review of Plant Biology 70 (2019): 321–346. 10.1146/annurev-arplant-050718-095919 30786235

[3] C. Delker , M. Quint , and P. A. Wigge , “Recent Advances in Understanding Thermomorphogenesis Signaling,” Current Opinion in Plant Biology 68 (2022): 102231. 35636376 10.1016/j.pbi.2022.102231

[4] Z. Chen , M. Galli , and A. Gallavotti , “Mechanisms of Temperature‐Regulated Growth and Thermotolerance in Crop Species,” Current Opinion in Plant Biology 65 (2022): 102134. 34749068 10.1016/j.pbi.2021.102134

[5] Y. H. Xing , H. Y. Lu , X. F. Zhu , et al., “How Rice Responds to Temperature Changes and Defeats Heat Stress,” Rice 17 (2024): 73. 39611857 10.1186/s12284-024-00748-2 PMC11607370

[6] Y. Zhou , F. Xu , Y. Shao , and J. He , “Regulatory Mechanisms of Heat Stress Response and Thermomorphogenesis in Plants,” Plants (Basel) 11 (2022): 3410. 36559522 10.3390/plants11243410 PMC9788449

[7] H. Zhang , Y. Zhao , and J. K. Zhu , “Thriving Under Stress: How Plants Balance Growth and the Stress Response,” Developmental Cell 55 (2020): 529–543. 33290694 10.1016/j.devcel.2020.10.012

[8] B. Zhang , S. Wu , Y. Zhang , et al., “A High Temperature‐Dependent Mitochondrial Lipase EXTRA GLUME1 Promotes Floral Phenotypic Robustness Against Temperature Fluctuation in Rice (*Oryza sativa* L.),” PLOS Genetics 12 (2016): 1006152. 10.1371/journal.pgen.1006152 PMC4930220 27367609

[9] D. Wang , B. Qin , X. Li , et al., “Nucleolar DEAD‐Box RNA Helicase TOGR1 Regulates Thermotolerant Growth as a Pre‐rRNA Chaperone in Rice,” PLOS Genetics 12 (2016): 1005844. 10.1371/journal.pgen.1005844 PMC4743921 26848586

[10] X. T. Li , H. S. Tang , T. Xu , et al., “N‐Terminal Acetylation Orchestrates Glycolate‐Mediated ROS Homeostasis to Promote Rice Thermoresponsive Growth,” New Phytologist 243 (2024): 1742–1757. 38934055 10.1111/nph.19928

[11] L. Z. Huang , M. Zhou , Y. F. Ding , and C. Zhu , “Gene Networks Involved in Plant Heat Stress Response and Tolerance,” International Journal of Molecular Sciences 23 (2022): 11970. 36233272 10.3390/ijms231911970 PMC9569452

[12] A. Ciechanover , “Proteolysis: From the Lysosome to Ubiquitin and the Proteasome,” Nature Reviews Molecular Cell Biology 6 (2005): 79–87. 15688069 10.1038/nrm1552

[13] S. L. Stone , “The Role of Ubiquitin and the 26S Proteasome in Plant Abiotic Stress Signaling,” Frontiers in Plant Science 5 (2014): 135. 24795732 10.3389/fpls.2014.00135 PMC3997020

[14] R. S. Marshall and R. D. Vierstra , “Dynamic Regulation of the 26S Proteasome: From Synthesis to Degradation,” Frontiers in Molecular Biosciences 6 (2019): 40. 31231659 10.3389/fmolb.2019.00040 PMC6568242

[15] D. Han , Z. Yu , J. Lai , and C. Yang , “Post‐Translational Modification: A Strategic Response to High Temperature in Plants,” aBIOTECH 3 (2022): 49–64. 36304199 10.1007/s42994-021-00067-w PMC9590526

[16] X. M. Li , D. Y. Chao , Y. Wu , et al., “Natural Alleles of a Proteasome α2 Subunit Gene Contribute to Thermotolerance and Adaptation of African Rice,” Nature Genetics 47 (2015): 827–833. 25985140 10.1038/ng.3305

[17] H. X. Yu , Y. J. Cao , Y. B. Yang , et al., “A TT1–SCE1 Module Integrates Ubiquitination and SUMOylation to Regulate Heat Tolerance in Rice,” Molecular Plant 17 (2024): 1899–1918. 39552084 10.1016/j.molp.2024.11.007

[18] J. Kurepa and J. A. Smalle , “Structure, Function and Regulation of Plant Proteasomes,” Biochimie 90 (2008): 324–335. 17825468 10.1016/j.biochi.2007.07.019

[19] P. Jeandet , M. Formela‐Luboinska , M. Labudda , and I. Morkunas , “The Role of Sugars in Plant Responses to Stress and Their Regulatory Function During Development,” International Journal of Molecular Sciences 23 (2022): 5161. 35563551 10.3390/ijms23095161 PMC9099517

[20] Y. L. Ruan , “Sucrose Metabolism: Gateway to Diverse Carbon Use and Sugar Signaling,” Annual Review of Plant Biology 65 (2014): 33–67. 10.1146/annurev-arplant-050213-040251 24579990

[21] A. A. Saddhe , R. Manuka , and S. Penna , “Plant Sugars: Homeostasis and Transport Under Abiotic Stress in Plants,” Physiologia Plantarum 171 (2021): 739–755. 33215734 10.1111/ppl.13283

[22] P. Salvi , R. Agarrwal , N. G. Kajal , M. Manna , H. Kaur , and R. Deshmukh , “Sugar Transporters and Their Molecular Tradeoffs During Abiotic Stress Responses in Plants,” Physiologia Plantarum 174 (2022): 13652. 10.1111/ppl.13652 35174495

[23] K. Yamada and Y. Osakabe , “Sugar Compartmentation as an Environmental Stress Adaptation Strategy in Plants,” Seminars in Cell & Developmental Biology 83 (2018): 106–114. 29287835 10.1016/j.semcdb.2017.12.015

[24] T. Gautam , M. Dutta , V. Jaiswal , G. Zinta , V. Gahlaut , and S. Kumar , “Emerging Roles of SWEET Sugar Transporters in Plant Development and Abiotic Stress Responses,” Cells 11 (2022): 1303. 35455982 10.3390/cells11081303 PMC9031177

[25] M. Miyazaki , M. Araki , K. Okamura , Y. Ishibashi , T. Yuasa , and M. Iwaya‐Inoue , “Assimilate Translocation and Expression of Sucrose Transporter, OsSUT1, Contribute to High‐Performance Ripening Under Heat Stress in the Heat‐Tolerant Rice Cultivar Genkitsukushi,” Journal of Plant Physiology 170 (2013): 1579–1584. 23910376 10.1016/j.jplph.2013.06.011

[26] S. Morita and H. Nakano , “Nonstructural Carbohydrate Content in the Stem at Full Heading Contributes to High Performance of Ripening in Heat‐Tolerant Rice Cultivar Nikomaru,” Crop Science 51 (2011): 818–828.

[27] G. G. Lehretz , S. Sonnewald , N. Lugassi , D. Granot , and U. Sonnewald , “Future‐Proofing Potato for Drought and Heat Tolerance by Overexpression of Hexokinase and SP6A,” Frontiers in Plant Science 11 (2021): 614534. 33510758 10.3389/fpls.2020.614534 PMC7835534

[28] M. Sharma , Z. Z. Banday , B. N. Shukla , and A. Laxmi , “Glucose‐Regulated HLP1 Acts as a Key Molecule in Governing Thermomemory,” Plant Physiology 180 (2019): 1081–1100. 30890662 10.1104/pp.18.01371 PMC6548265

[29] D. Granot and G. Kelly , “Evolution of Guard‐Cell Theories: The Story of Sugars,” Trends in Plant Science 24 (2019): 507–518. 30862392 10.1016/j.tplants.2019.02.009

[30] D. M. Daloso , L. Dos Anjos , and A. R. Fernie , “Roles of Sucrose in Guard Cell Regulation,” New Phytologist 211 (2016): 809–818. 27060199 10.1111/nph.13950

[31] F. Adolf , J. L. Du , E. A. Goodall , et al., “Visualizing Chaperone‐Mediated Multistep Assembly of the Human 20S Proteasome,” Nature Structural & Molecular Biology 31 (2024): 1176–1188. 10.1038/s41594-024-01268-9 PMC11327110 38600324

[32] K. Ferrell , C. R. M. Wilkinson , W. Dubiel , and C. Gordon , “Regulatory Subunit Interactions of the 26S Proteasome, a Complex Problem,” Trends in Biochemical Sciences 25 (2000): 83–88. 10664589 10.1016/s0968-0004(99)01529-7

[33] X. L. Huang , B. Luan , J. P. Wu , and Y. G. Shi , “An Atomic Structure of the human 26S Proteasome,” Nature Structural & Molecular Biology 23 (2016): 778–785. 10.1038/nsmb.3273 27428775

[34] H. X. Zhang , C. Y. Zhou , Z. Mohammad , and J. H. Zhao , “Structural Basis of Human 20S Proteasome Biogenesis,” Nature Communications 15 (2024): 8184. 10.1038/s41467-024-52513-0 PMC11410832 39294158

[35] K. Chen , T. Guo , X. M. Li , et al., “Translational Regulation of Plant Response to High Temperature by a Dual‐Function tRNAHis Guanylyltransferase in Rice,” Molecular Plant 12 (2019): 1123–1142. 31075443 10.1016/j.molp.2019.04.012

[36] J. H. Yang , X. Qu , L. Ji , et al., “PIF4 Promotes Expression of HSFA2 to Enhance Basal Thermotolerance in Arabidopsis,” International Journal of Molecular Sciences 23 (2022): 6017. 35682701 10.3390/ijms23116017 PMC9181434

[37] Z. Chen , Y. W. Huang , W. J. Yang , et al., “The Hydrogen Sulfide Signal Enhances Seed Germination Tolerance to High Temperatures by Retaining Nuclear COP1 for HY5 Degradation,” Plant Science 285 (2019): 34–43. 31203892 10.1016/j.plantsci.2019.04.024

[38] Y. L. Zhao , S. Liu , K. F. Yang , X. L. Hu , and H. F. Jiang , “Fine Control of Growth and Thermotolerance in the Plant Response to Heat Stress,” Journal of Integrative Agriculture 24 (2025): 409–428.

[39] N. B. Jensen , O. Vrobel , N. A. Nageshbabu , et al., “Stomatal Effects and ABA Metabolism Mediate Differential Regulation of Leaf and Flower Cooling in Tomato Cultivars Exposed to Heat and Drought Stress,” Journal of Experimental Botany 75 (2024): 2156–2175. 38207009 10.1093/jxb/erad498

[40] R. M. Marchin , B. E. Medlyn , M. G. Tjoelker , and D. S. Ellsworth , “Decoupling Between Stomatal Conductance and Photosynthesis Occurs Under Extreme Heat in Broadleaf Tree Species Regardless of Water Access,” Global Change Biology 29 (2023): 6319–6335. 37698501 10.1111/gcb.16929

[41] C. Naujokat and S. Hoffmann , “Role and Function of the 26S Proteasome in Proliferation and Apoptosis,” Laboratory Investigation 82 (2002): 965–980. 12177235 10.1097/01.lab.0000022226.23741.37

[42] J. Ganapathy , K. A. Hand , and N. Shabek , “Analysis of 26S Proteasome Activity Across Arabidopsis Tissues,” Plants 13 (2024): 1696. 38931128 10.3390/plants13121696 PMC11207565

[43] R. Kandel , J. Jung , and S. Neal , “Proteotoxic Stress and the Ubiquitin Proteasome System,” Seminars in Cell & Developmental Biology 156 (2024): 107–120. 37734998 10.1016/j.semcdb.2023.08.002 PMC10807858

[44] W. Li , K. Yang , C. F. Hu , et al., “A Natural Gene On‐off System Confers Field Thermotolerance for Grain Quality and Yield in Rice,” Cell 188 (2025): 3661–3678. 40311617 10.1016/j.cell.2025.04.011

[45] A. J. Crawford , D. H. McLachlan , A. M. Hetherington , and K. A. Franklin , “High Temperature Exposure Increases Plant Cooling Capacity,” Current Biology 22 (2012): R396–R397. 22625853 10.1016/j.cub.2012.03.044

[46] H. Lin , Y. J. Chen , H. L. Zhang , P. L. Fu , and Z. X. Fan , “Stronger Cooling Effects of Transpiration and Leaf Physical Traits of Plants From a Hot Dry Habitat Than From a Hot Wet Habitat,” Functional Ecology 31 (2017): 2202–2211.

[47] N. D. Udeshi , T. Svinkina , P. Mertins , et al., “Refined Preparation and Use of Anti‐Diglycine Remnant (K‐ε‐GG) Antibody Enables Routine Quantification of 10,000s of Ubiquitination Sites in Single Proteomics Experiments,” Molecular & Cellular Proteomics 12 (2013): 825–831. 23266961 10.1074/mcp.O112.027094 PMC3591673

[48] T. Tian , Y. Liu , H. Y. Yan , et al., “agriGO v2.0: A GO Analysis Toolkit for the Agricultural Community, 2017 Update,” Nucleic Acids Research 45 (2017): W122–W129. 28472432 10.1093/nar/gkx382 PMC5793732

[49] T. T. Chen , X. Chen , S. S. Zhang , et al., “The Genome Sequence Archive Family: Toward Explosive Data Growth and Diverse Data Types,” Genomics, Proteomics & Bioinformatics 19 (2021): 578–583. 10.1016/j.gpb.2021.08.001 PMC9039563 34400360

[50] Y. B. Xue , Y. M. Bao , Z. Zhang , et al., “Database Resources of the National Genomics Data Center, China National Center for Bioinformation in 2022,” Nucleic Acids Research 50 (2022): D27–D38. 34718731 10.1093/nar/gkab951 PMC8728233

