# Supplementary material for: TOGR3, a Proteasome β4 Subunit, Orchestrates Sugar Homeostasis to Trade Off Growth and Thermotolerance in Rice
Source: Adv Sci (Weinh). 2026 Jan 21;13(18):e16395. doi: 10.1002/advs.202516395 (PMC13042809; doi:10.1002/advs.202516395)
Supplement: Supplementary file 1 — Supporting file 1: advs73909‐sup‐0001‐SuppMat.docx [file ADVS-13-e16395-s002.docx]

**TOGR3, a Proteasome β4 Subunit, Orchestrates Sugar Homeostasis to Trade off Growth and Thermotolerance in Rice**

Biyao Zhang, Xiaoyan Wu, Ting Xu, Feifei Guo, Xiaolu Shen, Cuiping Meng, Yanan Wang, Xue Han, Hong Zhao, Yongbiao Xue


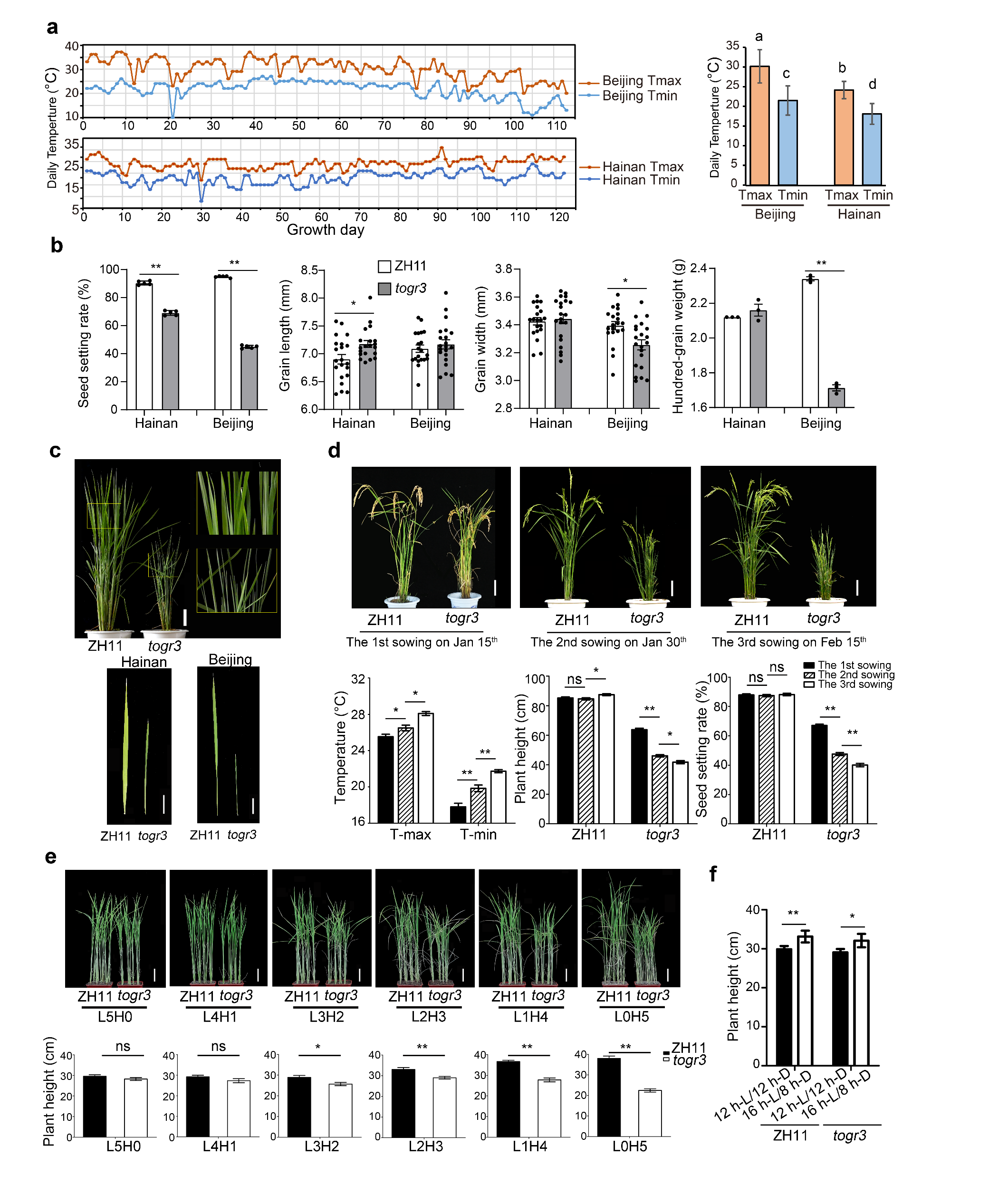


**Figure S1**. The *togr3* mutant exhibits impaired adaptive growth to high ambient temperature. a) Daily maximum and minimum temperatures during the rice-growing seasons in Hainan (winter) and Beijing (summer) in 2025. b) Phenotypic comparison of wildtype and *togr3* plants grown under natural field conditions in Hainan and Beijing. Seed setting rate (n = 5), hundred-grain weight (n = 3) and grain size (n ≥ 20) were quantified. c) Altered leaf morphology in *togr3*. d) Growth performance of wildtype and *togr3* plants across three sowing dates in Hainan. Corresponding daily temperature profiles are shown. Plant height (n ≥ 15) and seed setting rate (n ≥ 5) were evaluated. e) Temperature-dependent seedling growth response. Wildtype and *togr3* seedlings were grown under low (25/20°C) or high (35/30°C) temperatures for 5 weeks with different treatment regimens (e.g., L4H1 indicates 4 weeks at low temperature followed by 1 week at high temperature, n ≥ 15). f) Photoperiod response analysis. Plants were grown for 4 weeks at 25/20°C under short-day (12-h light/12-h dark) or long-day (16-h light/8-h dark) conditions. Data: mean ± SEM, assessed by the two-sided Student’s *t*-test with *P < 0.05, **P < 0.01.


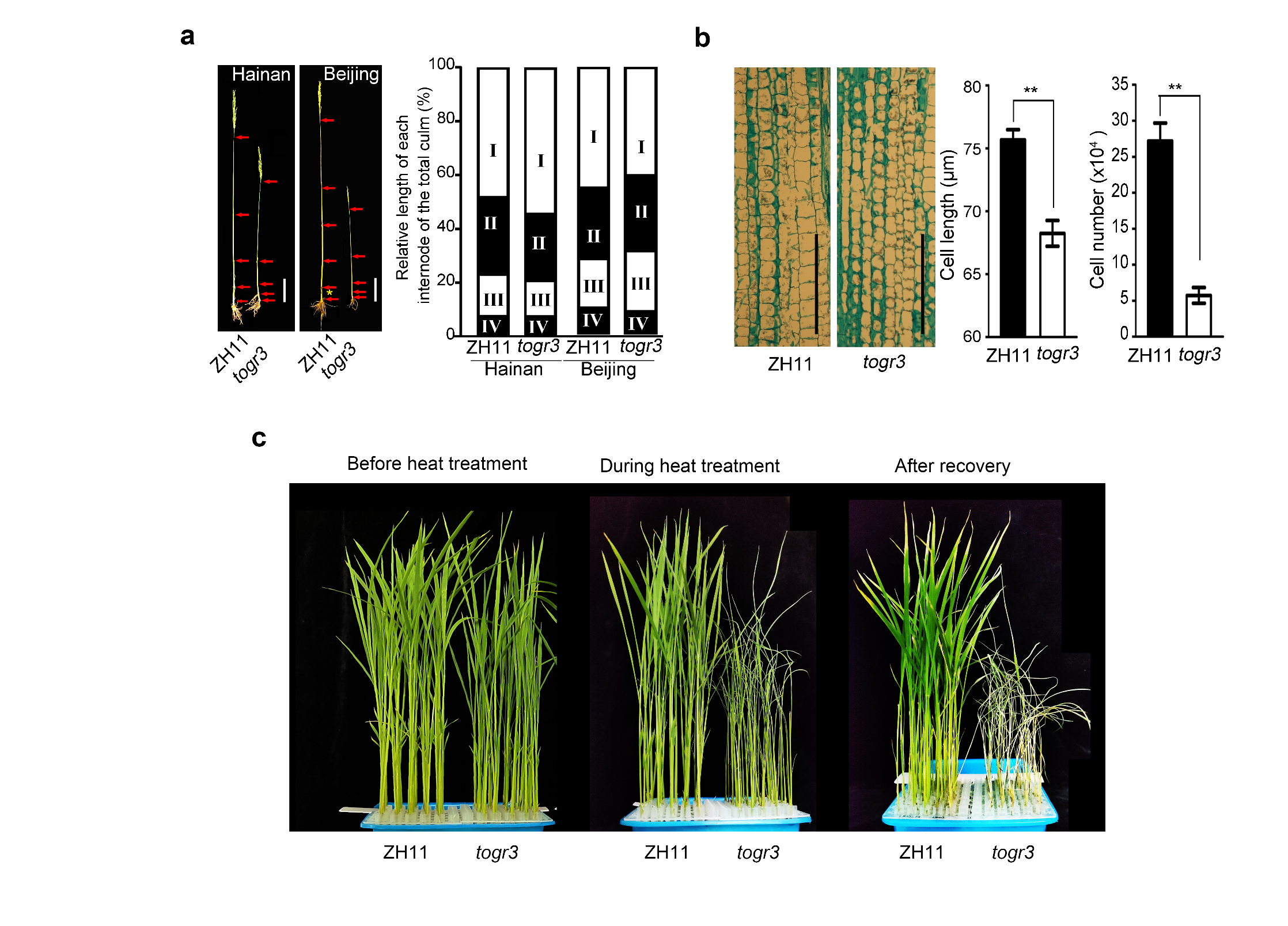


**Figure S2**. Anatomical and physiological characterization of *togr3*. a) Comparative internode morphology of wildtype and *togr3* plants under two environments. Internodes were numbered from the apex (I) to the base (IV); red arrowheads indicate stem nodes. Bar graph shows the relative length of each internode (n = 12). b) Cellular basis of internode shortening. Cell length and cell number in the second internode of Beijing-grown plants were quantified (n ≥ 500). c) Rapid desiccation phenotype of *togr3* seedlings during heat treatment. Data shown as mean ± SEM (two-sided Student’s *t*-test: *P < 0.05, **P < 0.01) in (b). Scale bars: 10 cm (a); 500 μm (b).


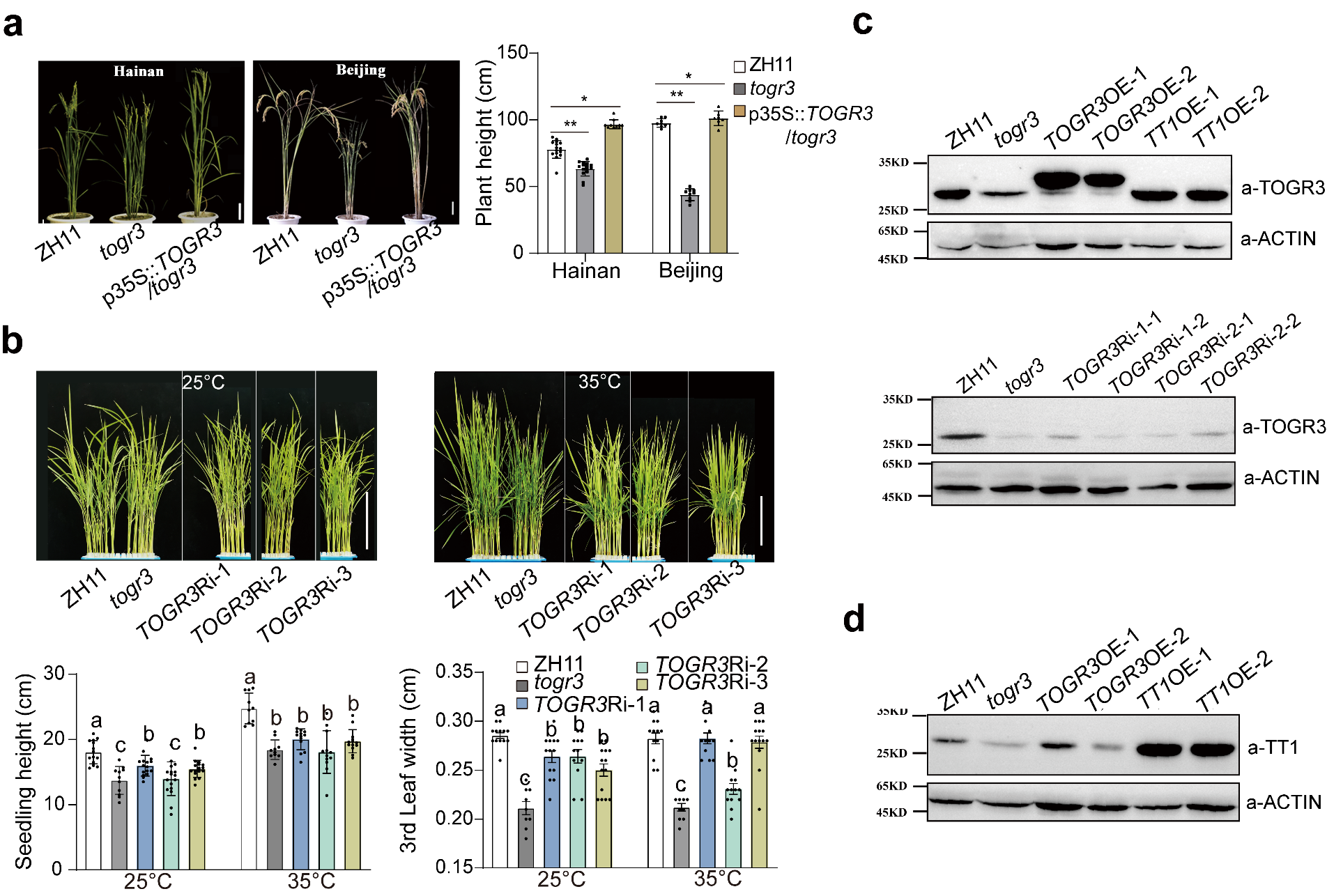


**Figure S3**. Functional validation of TOGR3 as the 26S proteasome β4 subunit. a) Genetic complementation of *togr3* with *p35S*::*TOGR3* (n=15). b) RNA interfering (RNAi) analysis of *TOGR3*. Seedling height was analyzed in three *TOGR3* RNAi lines grown under moderate and high temperatures (n ≥ 15). c,d) Immunoblot detection of TOGR3 and TT1 proteins in overexpression or RNAi lines. *TOGR3*-3x*FLAG* and *TT1^CG14^* were overexpressed in the ZH11 background. Data represent mean ± SEM and significances were assessed by the two-sided Student’s *t*-test with *P < 0.05, **P < 0.01 (a) and Duncan’s multiple range test (b). Scale bars: 10 cm (a, b).


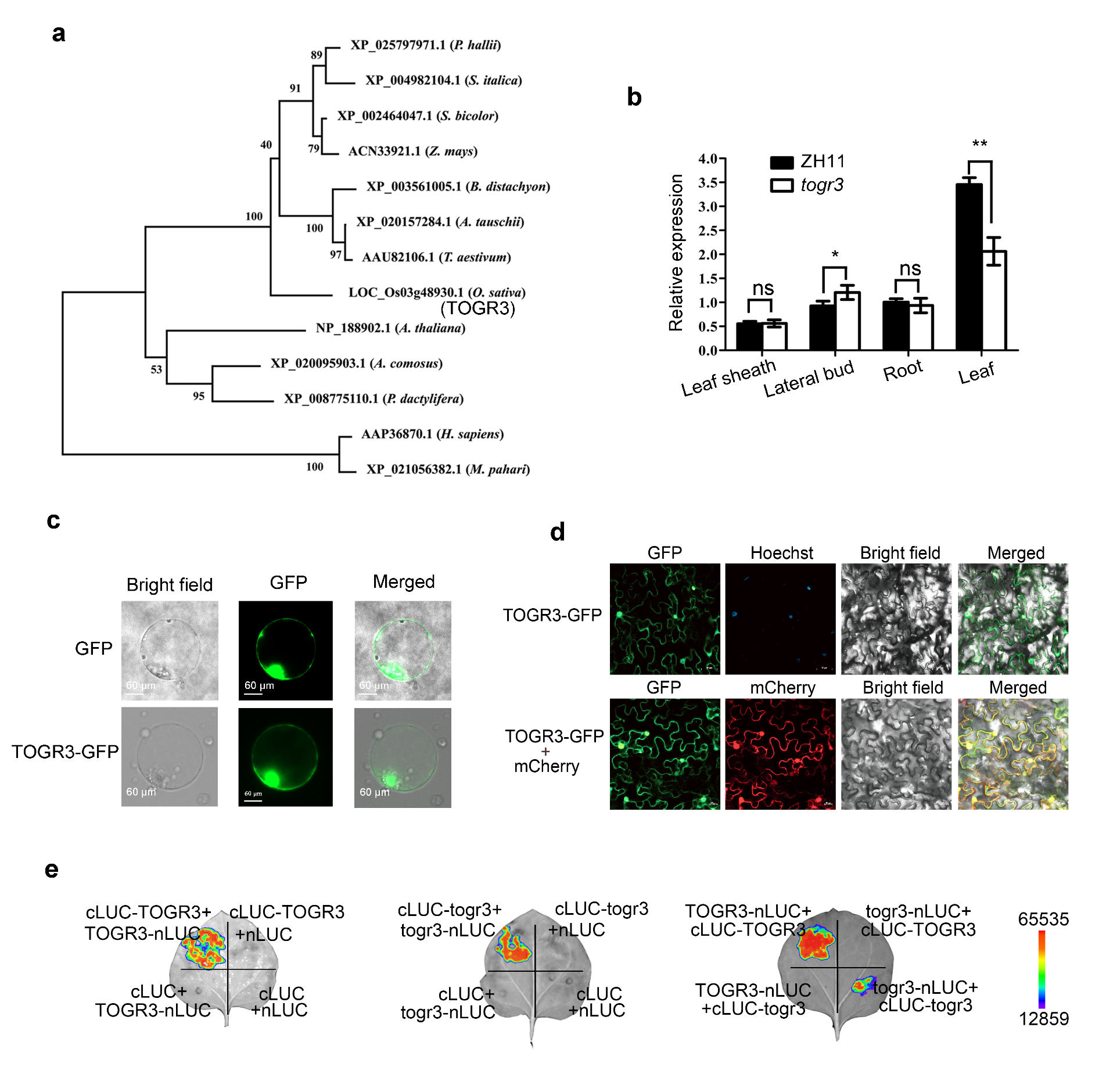


**Figure S4.** *TOGR3* encodes a conserved 26S proteasome β4 subunit localized in nucleus and cytoplasm. a) Phylogenetic conservation of the 26S proteasome β4 subunit across eukaryotes. b) Tissue-specific expression of *TOGR3* in wildtype and *togr3* seedlings. c) Subcellular localization of TOGR3–GFP in rice protoplasts. d) Subcellular localization of TOGR3–GFP in tobacco epidermal cells. GFP signals were merged with nuclear dye (Hoechst) or a nucleus–cytoplasm marker (mCherry). e) Luciferase complementation imaging assay in tobacco leaves. Data represent mean ± SEM; significance was assessed by two-sided Student’s t-test (*P < 0.05, **P < 0.01). Scale bars: 60 μm (c), 50 μm (d).


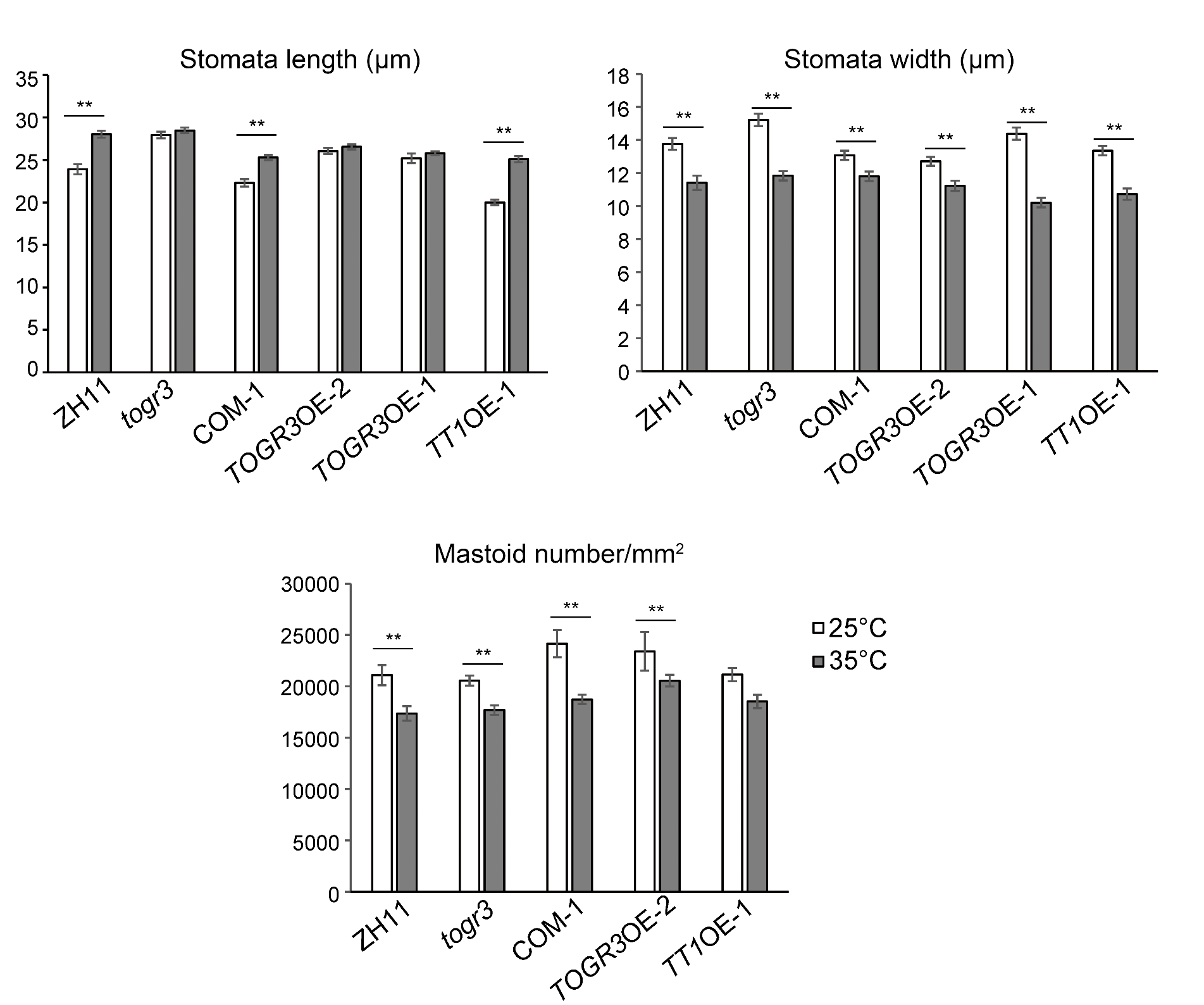


**Figure S5.** Quantitative analysis of stomatal morphology. Stomata length and width (n = 50) and mastoid density (n = 10) were analyzed in ZH11, *togr3*, *TOGR3* complementary (COM), and *TOGR3*/*TT1* overexpression (OE) plants. Data represent mean ± SEM and significances were assessed by the two-sided Student’s t-test with *P < 0.05, **P < 0.01.


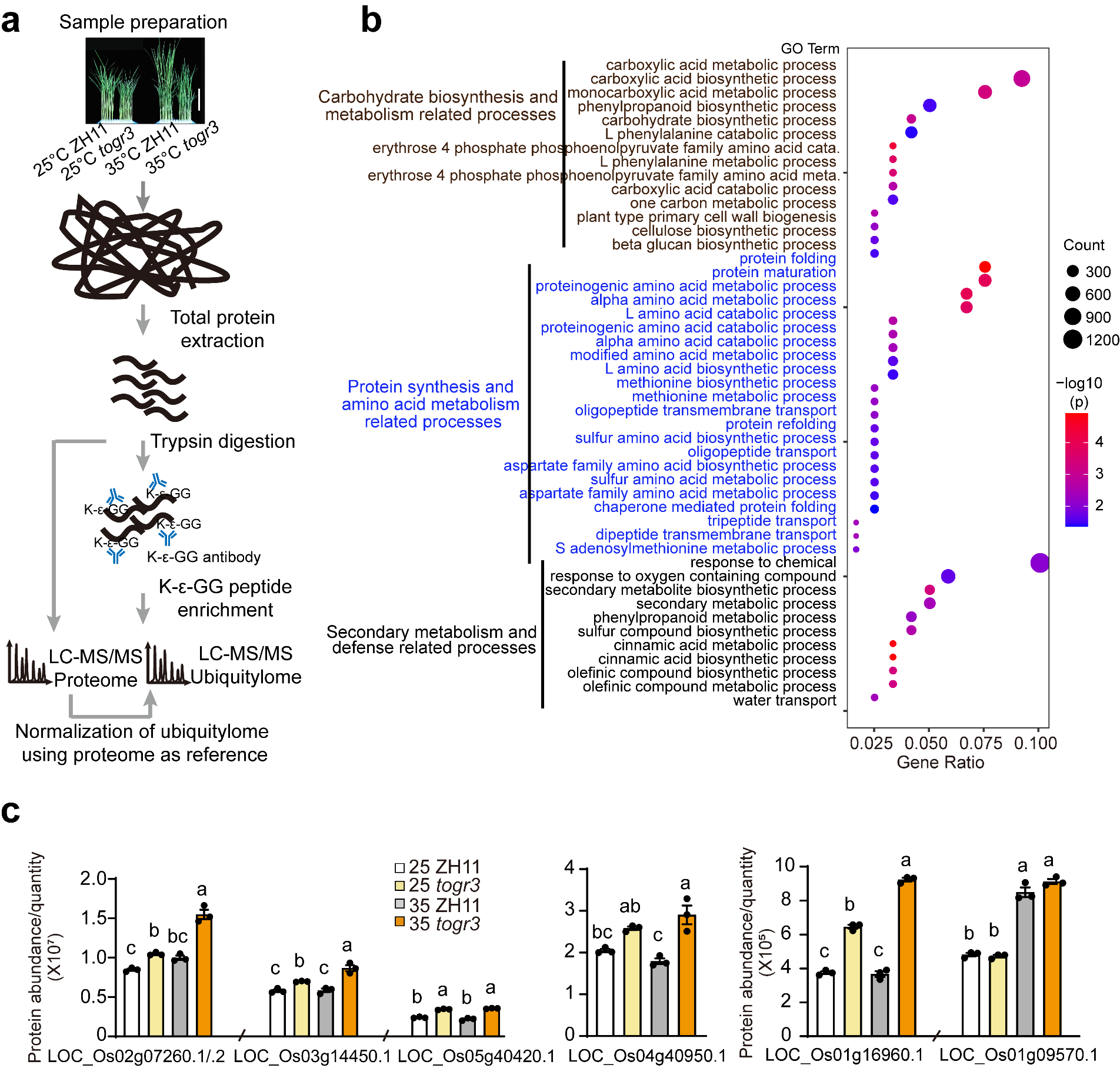


**Figure S6.** Thermoresponsive ubiquitylome profiling. a) Schematic overview of ubiquitin-modified proteome analysis. Leaves from 2-week-old wildtype and *togr3* seedlings grown at 25/20 °C or 35/30 °C were analyzed. Synchronous proteome data were used as internal references. Three biological replicates were performed. b) GO biological processes of differentially ubiquitinated proteins associated with TT1 (data adapted from Li *et al.*, 2015)[16] c) Protein abundance of six carbohydrate metabolism enzymes detected by proteomics. Data represent mean ± SEM and assessed by Duncan’s multiple range test.


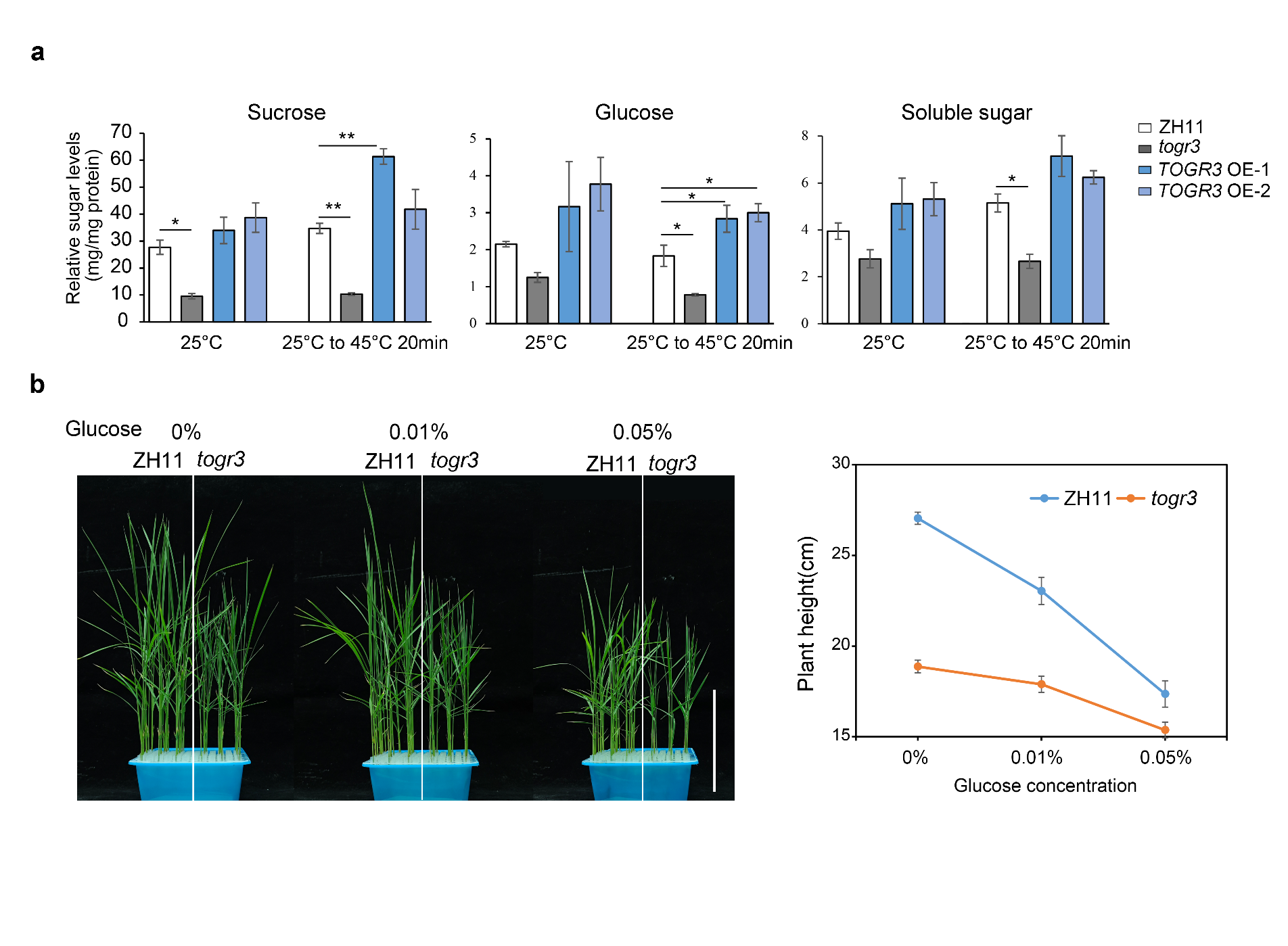


**Figure S7.** *TOGR3* regulates sugar metabolism and signaling under heat stress. a) Relative levels of sucrose, glucose, and total soluble sugars (normalized to protein) in ZH11, *togr3*, and *TOGR3* overexpression seedlings (n = 3). b) Seedling height of wildtype and *togr3* grown at 35/30°C for two weeks in media supplemented with the indicated glucose concentrations (w/v). Data represent mean ± SEM; significances were assessed by two-sided Student’s *t*-test (*P < 0.05, **P < 0.01) in (a). Scale bar: 10 cm.


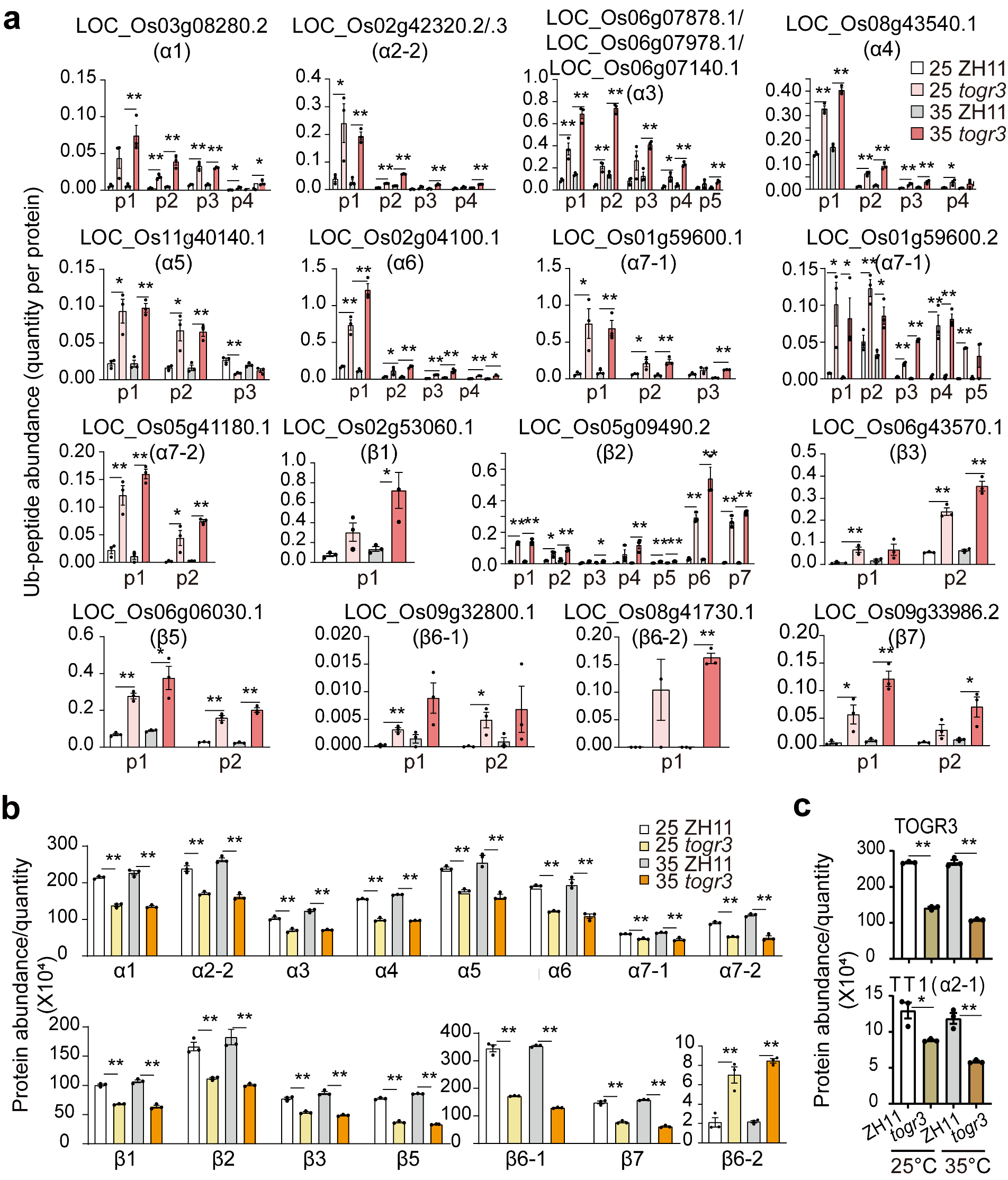


**Figure S8.** *TOGR3* promotes self-recycling of the 26S proteasome core particle. a) Relative abundance of ubiquitinated peptides from rice 26S proteasome core subunits, identified based on Arabidopsis homologs. Ubiquitylome data were normalized against proteome levels. b) Protein abundance of 26S proteasome core subunits (proteomic data). c) Protein abundance of TOGR3 and TT1. Data represent mean ± SEM; significances were assessed by two-sided Student’s *t*-test (*P < 0.05, **P < 0.01).


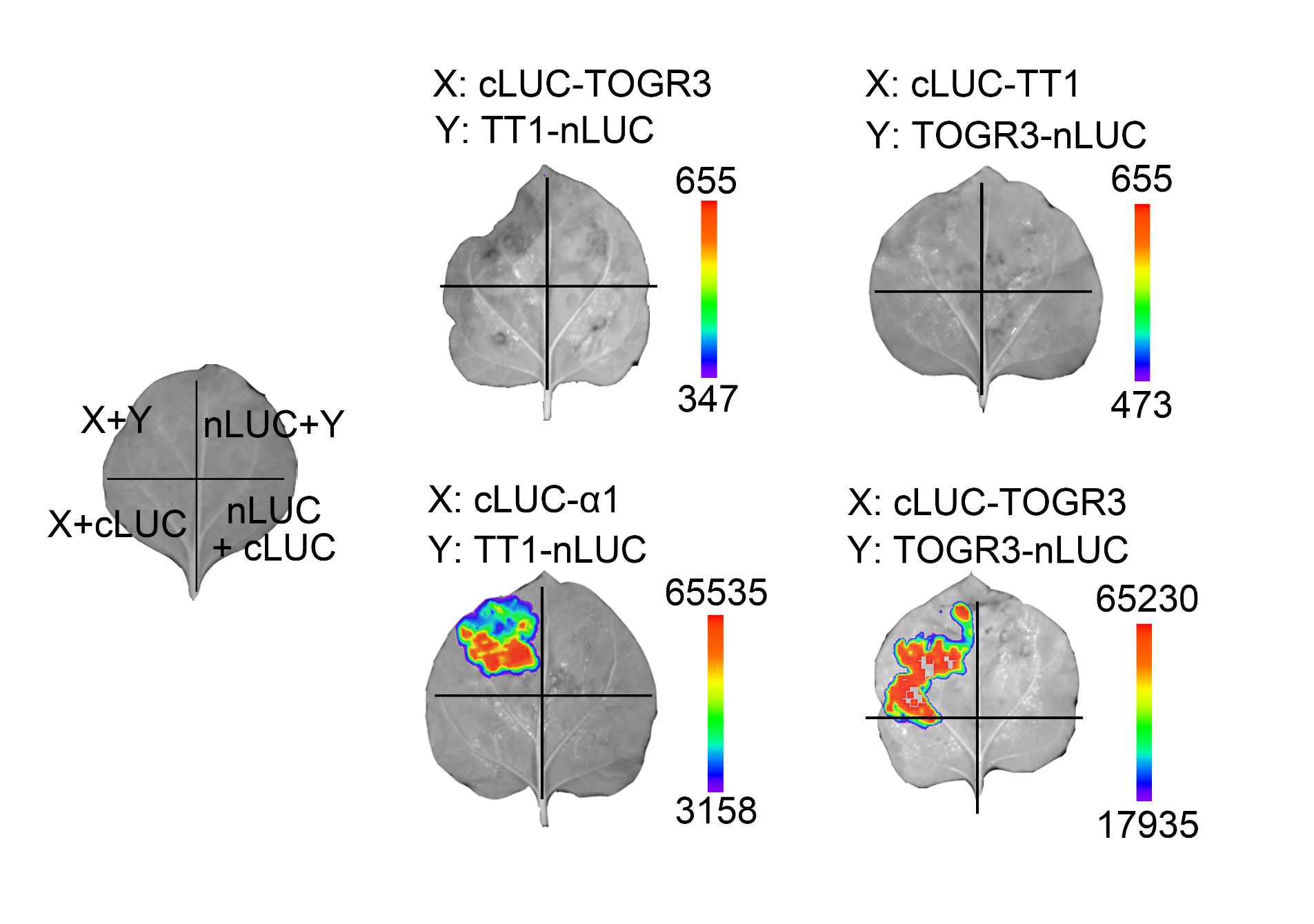


**Figure S9.** TOGR3 does not directly interact with TT1. Luciferase complementation imaging assays in tobacco leaves show no direct interaction between TOGR3 and TT1. α1 (LOC_Os03g08280.2)–α2 (TT1) and β4 (TOGR4)–β4 interactions served as positive controls.
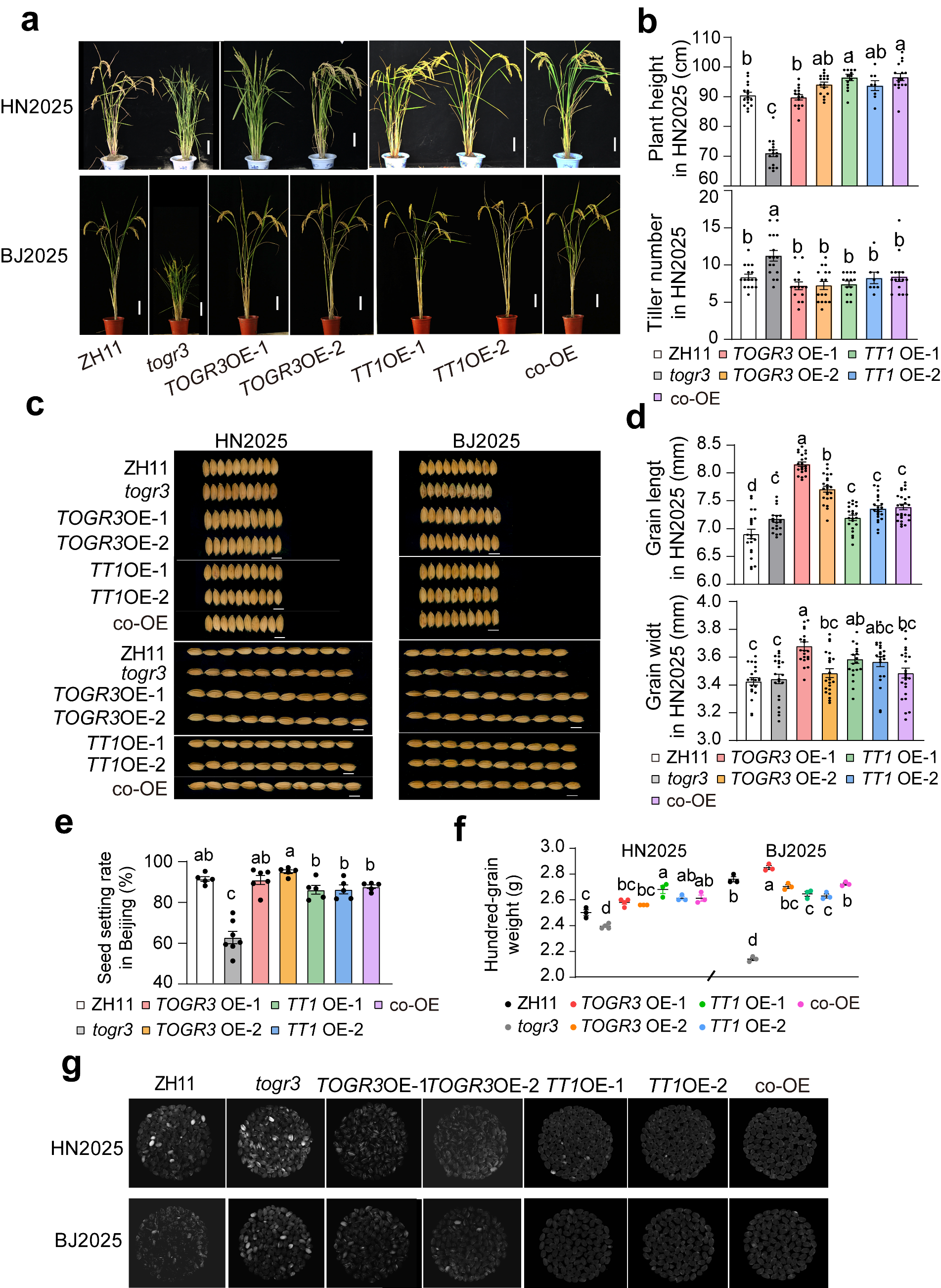


**Figure S10.** *TOGR3–TT1* co-overexpression stabilizes grain yield and quality under high temperature.

Agronomic traits—including plant architecture (a, b), grain size (c, d), seed setting rate (e), grain weight (f), and grain quality (g)—were evaluated in ZH11, *togr3*, *TOGR3*-OE, *TT1*-OE, and *TOGR3–TT1* co-OE plants grown under natural field conditions in Hainan winter and Beijing summer (2025). Data collection and sample sizes are as described in Figure 5. Data: mean ± SEM; analyzed by Duncan’s test (P < 0.05). Scale bars: 10 cm (a), and 5 mm (c).

Table S1. Annotation of All Detected Ubiquitin-Modified Peptides in Ubiquitylome.

Table S2. Comparisons of Ubiquitin-Modified Proteins between Samples.

Table S3. Primers Used in the Paper.

Table S4. Main Figure Data Source.

Table S5. Supplemental Figure Data Source.
